# Insertion of Badnaviral DNA in the Late Blight Resistance Gene (R1a) of Brinjal Eggplant (*Solanum melongena*)

**DOI:** 10.3389/fpls.2021.683681

**Published:** 2021-07-23

**Authors:** Saad Serfraz, Vikas Sharma, Florian Maumus, Xavier Aubriot, Andrew D. W. Geering, Pierre-Yves Teycheney

**Affiliations:** ^1^CIRAD, UMR AGAP Institut, F-97130, Capesterre-Belle-Eau, France; ^2^UMR AGAP Institut, Univ Montpellier, CIRAD, INRAE, Institut Agro, Capesterre-Belle-Eau, France; ^3^Centre of Agricultural Biochemistry and Biotechnology, University of Agriculture, Faisalabad, Pakistan; ^4^URGI, INRAE, Université Paris-Saclay, Versailles, France; ^5^Université Paris-Saclay, CNRS, AgroParisTech, Ecologie Systématique Evolution, Orsay, France; ^6^Queensland Alliance for Agriculture and Food Innovation, The University of Queensland, Brisbane, QLD, Australia

**Keywords:** Endogenous viral elements, badnavirus, *Solanum melongena*, *R1* gene, co-transcripts, phylogeny

## Abstract

Endogenous viral elements (EVEs) are widespread in plant genomes. They result from the random integration of viral sequences into host plant genomes by horizontal DNA transfer and have the potential to alter host gene expression. We performed a large-scale search for co-transcripts including caulimovirid and plant sequences in 1,678 plant and 230 algal species and characterized 50 co-transcripts in 45 distinct plant species belonging to lycophytes, ferns, gymnosperms and angiosperms. We found that insertion of badnavirus EVEs along with Ty-1 copia mobile elements occurred into a late blight resistance gene (*R1*) of brinjal eggplant (*Solanum melongena*) and wild relatives in genus *Solanum* and disrupted *R1* orthologs. EVEs of two previously unreported badnaviruses were identified in the genome of *S. melongena*, whereas EVEs from an additional novel badnavirus were identified in the genome of *S. aethiopicum*, the cultivated scarlet eggplant. Insertion of these viruses in the ancestral lineages of the direct wild relatives of the eggplant would have occurred during the last 3 Myr, further supporting the distinctiveness of the group of the eggplant within the giant genus *Solanum*.

## Introduction

Endogenous viral elements (EVEs) originate from viruses with RNA or DNA genomes. They are widespread in eukaryotic genomes (Holmes, 2011; Feschotte and Gilbert, 2012) and may comprise large portions of these genomes: it is estimated that 5–8% of the human genome is composed of endogenous retroviruses (ERVs; Holmes, 2011). Integration is active for viruses encoding an integrase, such as retroviruses, but for others without this protein, such as all plant viruses, integration is either passive through non-homologous end-joining (NHEJ) or mediated by retrotransposons (Geuking et al., 2009; Taylor and Bruenn, 2009; Horie et al., 2010). Some EVEs are infective but the majority are replication-defective because of sequence decay or because host regulation mechanisms have co-evolved to suppress their expression (Mitreiter et al., 1994; Feschotte and Gilbert, 2012).

Endogenous viral elements are important sources of novel genetic information that can ultimately play a significant role in the evolution of the host. For example, syncytin-A, a protein involved in placental development in mammals, is encoded by a gene that was acquired through the endogenization of retroviral *Env* gene (Sha et al., 2000; Dupressoir et al., 2005). ERVs can also modify host gene expression through the contribution of alternate promoters, aberrant splicing, premature termination of transcription or gene disruption (Meyer et al., 2017). In plants, the acquisition of novel functions through the endogenization of viral genes has not yet been formally demonstrated. However, Mushegian and Elena (2015) reported the presence of genes encoding homologs to plant virus movement proteins (MPs) from the 30K superfamily in the genome of almost all euphyllophyte plants and their transcription into mRNAs. They also showed that several of the MP-like coding genes experience positive selection at the codon level, suggesting these genes might be expressed and serve a function in plants (Mushegian and Elena, 2015). Filloux et al. (2015) reported the discovery of endogenous geminivirus like sequences (EGV1) in the genome of *Dioscorea* spp. of the *Enantiophyllum* clade. They provided evidence that functional EGV-expressed replication-associated protein (Rep) were expressed in yams for extended periods following endogenization and that some of them are possibly still functionally expressed in several species of the *Enantiophyllum* clade (Filloux et al., 2015).

In plants, most characterized EVEs originate from viruses with DNA genomes in the families *Caulimoviridae* and *Geminiviridae* (Teycheney and Geering, 2011; Geering et al., 2014; Filloux et al., 2015; Diop et al., 2018; Sharma et al., 2020). Caulimovirid EVEs are widespread in all major plant taxa including club mosses, ferns, gymnosperms and angiosperms (Geering et al., 2014; Diop et al., 2018; Gong and Han, 2018). Only five infective EVEs have been reported in plants. All originate from viruses in the family *Caulimoviridae* (Ndowora et al., 1999; Lockhart et al., 2000; Richert-Pöggeler et al., 2003: Chabannes et al., 2013). It is assumed that non-infectious caulimovirid EVEs must impart some beneficial functions to the plant to be retained over periods as long as millions of years. It has been hypothesized that caulimovirid EVEs confer resistance to cognate exogenous viruses by acting as natural viral transgenes to prime gene silencing either at transcriptional or post-transcriptional levels (Mette et al., 2002; Teycheney and Tepper, 2007; Bertsch et al., 2009). However, copy number often far exceeds that necessary to produce efficient gene silencing and there are very few examples where the corresponding exogenous virus is still extant, making resistance a somewhat null function.

Research on the role of caulimovirid EVEs in normal plant metabolism is still very limited. Endogenous caulimovirid movement protein (MP) genes have been reported in most vascular plants that have had their genomes sequenced (Mushegian and Elena, 2015). There is experimental evidence that downregulation of an endogenous MP in the model plant *Arabidopsis thaliana* can result in a small delay in plant development, and reduces the germination rate, especially at high salt concentrations (Carrasco et al., 2019). In another case study, activation of endogenous petunia vein clearing virus (PVCV) to produce independently replicating virus leads to suppression of post-transcriptional silencing of a gene in the anthocyanin biosynthesis pathway and development of pink blotches in the normally white sections of the bicolored petunia flower (Kuriyama et al., 2020). Impacts of endogenization of viral sequences on genome plasticity, host gene expression or gene disruption through insertional mutagenesis are not yet documented in plants but they are likely to occur considering that such impacts have been reported repeatedly for transposable elements (Bennetzen, 2000; Martin et al., 2009; Zeng and Cheng, 2014).

In this study, we investigated the role of caulimovirid EVEs in plant gene expression through a systematic search for co-transcripts including host and caulimovirid sequences. We provide evidence for such co-transcripts in 45 plant species spanning lycophytes, ferns, gymnosperms and angiosperms. We found that the insertion of badnavirus EVEs resulted in the disruption of orthologs of late blight resistance genes (*R1*) in the cultivated brinjal eggplant (*Solanum melongena*) and its direct relatives. This viral insertion promoted alternative splicing of the co-transcripts and provided an alternate viral promoter. We also report on the identification of three novel badnaviruses for which complete genomes were assembled from EVEs whose insertion was estimated to have occurred during the last 3 million years (Myr).

## Materials and Methods

### Transcriptome Analysis

A search for co-transcripts encompassing viral and plant genomic sequences was done using BLASTX on 939 plant and 13 algal transcriptome shotgun assemblies (TSA) available at NCBI (Supplementary Table 1). In addition, 1,468 transcriptome assemblies from 943 plant and 230 algal species from the OneKP database^1^ were also included in the transcriptome dataset (Supplementary Table 1; Leebens-Mack et al., 2019).

Seventy-two complete genome sequences from viruses in eight genera of family *Caulimoviridae* (*Badnavirus, Caulimovirus, Cavemovirus, Petuvirus, Rosadnavirus, Solendovirus, Soymovirus, Tungrovirus*; Teycheney et al., 2020) and in the tentative genus Florendovirus (Geering et al., 2014) served as queries. tBLASTx-based search was performed using default parameters (except *E* value of 10–5). A library of LTR-retrotransposon sequences was used to filter tBLASTx hits and remove retrotransposon sequences. Transcripts were then annotated using conserved domain search (CDD) database (Lu et al., 2020^2^). An R-based script was designed and used to screen annotated transcripts for the presence of both viral and host domains. All co-transcripts were verified manually using NCBI’s CDD browser (Team R Development Core, 2018; Lu et al., 2020). Complete annotated transcripts were aligned and visualized using Easyfig V-2.2.2 (^3^ Sullivan et al., 2011). Nucleotide and protein sequence alignments were utilized to extract codon alignments using PAL2NAL program (Suyama et al., 2006). The Fast unbiased Bayesian approximation (FUBAR) method was implemented to compute dN/dS ratio from codon alignments (Weaver et al., 2018).

### Analysis of Alternative Splicing and Quantitative Gene Expression

Unassembled RNA-seq datasets for eggplant and related species (Page et al., 2019) were retrieved from sequence read archive (SRA: Supplementary Table 1). Adapter trimming and quality filtering was performed on all datasets. *Solanum melongena* reference genome sequence data (Barchi et al., 2019) was retrieved from the solgenomics server^4^. STAR (v-2.7.3a) was utilized to assemble the transcripts on *S. melongena* scaffold Ch0.05855 (Dobin et al., 2013). Alternative splicing finder (AS Finder; Min, 2013) was used for mapping alternative splicing sites on assembled transcripts. Two kbp sequences upstream each transcript were analyzed using Transcriptional Start Site Plant (TSSPlant), which predicts potential transcription start sites by combining characteristics describing functional motifs and oligonucleotide composition of these sites (Shahmuradov et al., 2017), and Neural Network Promoter Prediction (NNPP), which identifies eukaryotic and prokaryotic promoters in DNA sequences (Reese, 2001). Conserved motifs were identified using the plant *Cis-*Acting Regulatory Elements (CARE) database (Lescot et al., 2002).

Analyses of quantitative gene expression were performed on RNA-seq reads from *S. melongena* (two samples, including one under the name *S. ovigerum*, a primitive domesticate of the eggplant; see Page et al., 2019) as well as on two closely related wild species, *S. incanum* and *S. insanum.* RNA-seq reads were mapped on brinjal eggplant (*S. melongena*) genome scaffold Ch0-5855 using STAR aligner (Dobin et al., 2013). Alignment files and genomic scaffolds served as input for Cufflinks v2.2.1 (Trapnell et al., 2012). The resulting Gene Transfer Format (GTF) files generated from each RNA-seq dataset were merged using Cuffcompare script within the Cufflinks package (Trapnell et al., 2012). Output files were further processed by R-based package cummeRbund v 2.28 (Goff et al., 2012) to quantify the expression of genes and their transcript variants.

### Search for Endogenous Caulimovirid Sequences, Reconstruction of Complete Viral Genomes and Phylogenetic Analyses

We searched the genomes of *S. melongena* and the closely related species, *S. aethiopicum* (African scarlet eggplant), for caulimovirid reverse transcriptase (RT) protein sequences. For this, the RT-based protein library built by Diop et al. (2018) from 41 RT domains from distinct viruses representing all the genera in the family *Caulimoviridae* was used as queries for a tBLASTn-based search as described above. Putative integration loci were extended 5 kbp upstream and downstream of integration sites. Each extended locus was filtered for background LTR-retrotransposon sequences as described above. Filtered loci were translated using program translate (version 1.6) and protein sequences of 120 amino acids or more were compared to the initial RT library using BLASTp with default parameters (except *E* value 10^–5^). The resulting set of endogenous RT sequences was clustered using UCLUST (Edgar, 2010) with identity threshold set at 55%. Loci containing caulimovirid sequences were aligned with sequences from the initial RT library using MUSCLE (version 1.26; Hull, 2009). Maximum likelihood analysis was performed on RT-RNase H1 (RH1) regions using 1000 bootstrap values and phylogenetic trees were built using a 80% identity threshold to delineate viral species (Harrach et al., 2011). Phylogenetic trees were built using IQTree v.2.0, choosing the best-fit model of evolution (command -m GTR + F + I + G4) and 1000 bootstrap replicates (Nguyen et al., 2015). Dioscorea nummularia-associated virus (DNUaV) was used as an outgroup. Phylogenetic trees were also reconstructed using Bayesian inference methods. The best-fitting nucleotide substitution model for Bayesian inference was selected based on the Akaike information criterion computed by MrModeltest v.2.3 (Nylander et al., 2004); the GTR + G + I model was chosen and used in MrBayes 3.2.6 (Huelsenbeck and Ronquist, 2001). MrBayes analysis constituted two independent parallel runs of four Markov chains each, implemented for 5 million generations and sampled every 500 generations. Adequate mixing of the Markov chains and convergence of the two runs were confirmed with Tracer v1.6 (Rambaut et al., 2014). After removing a 10% burnin, the remaining trees were used to generate the 50% Bayesian majority-rule consensus tree. The reconstruction of complete badnavirus genomes from EVEs was carried out using previously described methods (Geering et al., 2014). The recombination detection program RDP v 4 (Martin et al., 2015) was used with default settings to detect recombination signals. Viral sequences were initially aligned by MUSCLE (version 1.26; Hull, 2009) and manually edited for recombination analysis.

### Synteny and Phylogenetic Analyses

*R1* ortholog loci identified in *S. aethiopicum*, *S. demissum*, *S. melongena*, *S. tuberosum*, and *S. verrucosum* were extended manually up to 15 kbp upstream and downstream. Reciprocal BLASTn analyses were performed on these extended loci. Synteny analyses were performed using Easyfig V-2.2.2 (Sullivan et al., 2011), with conserved genes located downstream *R1* genes as chromosomal anchors.

A library of NB-ARC domains was constructed from reference plant resistance (*R*) genes of the PRG database (Sanseverino et al., 2009) and used to search NB-ARC homologs in chromosomal contigs from *S. aethiopicum*, *S. commersonii*, *S. melongena*, *S. tuberosum*, and *S. verrucosum* using tBLASTn. Output sequences were translated using program translate (version 1.9) and aligned using MUSCLE. Resulting MSA files were used to build phylogenetic trees as described above except best-fit model of evolution (command – m JTT + F + R4).

A library of RT domains from transposable elements (TEs) was built by clustering reference TEs from repeat explorer database (Novák et al., 2013) at 50% identity threshold. This TE library served as queries to search each contig from *Solanum* species using tBLASTn. Phylogenetic comparisons of output TEs were performed as described above except best-fit model of evolution (command – m VT + F + G4). LTRHarvest was also utilized for LTR prediction and annotation (Ellinghaus et al., 2008).

### Plant Material, PCR Amplification and Sequencing

Seeds from 26 eggplant accessions and their wild relatives were provided by INRAE-Centre for Vegetable Germplasm (^5^ Supplementary Table 2). Seed-lines were selected to include nine of the 13 currently recognized species of the Eggplant clade, as delimited by Knapp et al. (2013) and Aubriot et al. (2018), including the wild progenitor of the eggplant, *S. insanum*. Seven species from the Anguivi grade (viz. a large and unresolved Afro-Asian lineage that includes the Eggplant clade and *S. aethiopicum*, the cultivated scarlet eggplant; see Aubriot et al., 2016) were also sampled (Knapp et al., 2013; Aubriot et al., 2016). Seeds were soaked for 24 h at room temperature in Petri dishes containing 500 ppm of gibberellic acid-3 (GA3), transferred to pots containing potting mix and let germinate at room temperature. Leaf samples were collected from seedlings at the five-leaf stage and stored at −80°C until further use. Total DNA was extracted using FastDNA kit (MP Bio, Irvine, CA, United States) and used to search for Caulimovirid EVE insertion loci by PCR using primer pair InL_F2 (CAAACAATACGGTACAACTC)/InL_R2 (CAACAGCATCGATGAATTC; Supplementary Table 3). These primers were designed based on the alignment of the flanking regions of the viral insertion locus of *S. melongena* (V*R1*) and the corresponding sequences in the genome of *S. aethiopicum*, which has no viral insertion. PCRs were done using Phusion DNA polymerase (NEB, Ipswich, MA, United States). PCR mixes with a final volume of 25 μl contained 2.5 μl of 10 × GC buffer, 0.2 μl of 25 mM MgCl_2_, 1.6 μl of 2.5 mM dNTPs, 1 U of 5,000 U/ml Phusion DNA polymerase and 5 μl of each primer at a concentration of 5 μM. Thermocycling conditions were an initial denaturation of 5 mn at 98°C, 35 cycles of 10 s at 98°C, 15 s at 63°C and 3 mn at 72°C followed by a final extension of 5 mn at 72°C. Amplification products were purified using QIAquick gel extraction kit (QIAGEN, Toronto, ON, Canada) and sequenced by Genewiz (Leipzig, Germany). In order to amplify upstream and downstream border regions, an additional set of primers (R1U: CTCGATTATGCCAAATCCTC and R1D: GCTTGAAGTATGCGAGAAG) was designed within the viral insertion of *S. melongena* (Supplementary Table 3). The left and right borders of the V*R1* insertion locus were amplified by PCR using the primer pairs InL_F2/R1U and lnLR2/R1D, respectively. PCR mixes with a final volume of 25 μl contained 2.5 μl of 10 × reaction buffer (10 mM Tris–HCl, 50 mM KCl, 1.5 mM MgCl_2_, pH 8.3), 0.75 μl of 25 mM MgCl_2_, 1 μl of 2.5 mM dNTPs, 1 U of 5,000 U/ml *Taq* DNA polymerase (NEB, Ipswich, MA, United States) and 2.5 μl of each primer at a concentration of 2 μM. PCR conditions were an initial denaturation of 5 mn at 94°C and 35 cycles of 15 s 94°C for, 30 s at 54°C and 2 mn at 72° followed by a final extension of 5 mn at 72°C. Amplification products were cloned using pGEM^®^-T Easy Vector Systems (Promega, Charbonnières-les-Bains, France) and sequenced by Genewiz (Leipzig, Germany).

## Results

### Search for Co-transcripts

**FIGURE 1:**
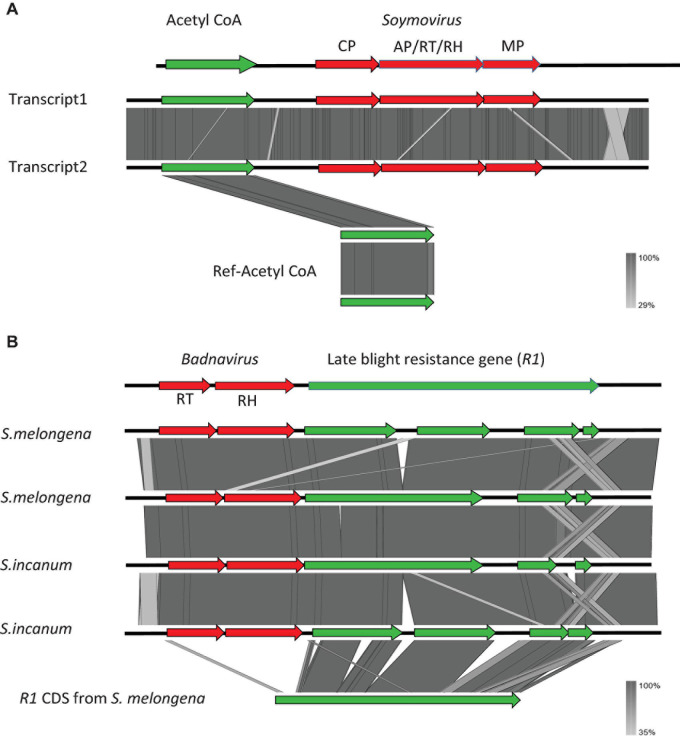
Structure of the co-transcripts identified in alfalfa (*Medicago sativa*) **(A)** and eggplant (*Solanum melongena*) and *Solanum incanum* **(B)**. Viral sequences are shown in red and host sequences are shown in green.

We used a tBLASTx-based pipeline to search the transcriptomes of 1,678 plant and 230 algal species for potential co-transcripts including host and caulimovirid sequences. Fifty distinct co-transcripts were identified from the transcriptomes of 45 plant species representing lycophytes, ferns, gymnosperms, and angiosperms (Table 1). These co-transcripts include sequences encoding partial or complete caulimovirid MP, reverse-transcriptase/ribonuclease H (RT-RH) or aspartic protease (AP). Co-transcripts including caulimovirid MPs and host chaperone sequences were identified in several distinct species (Table 1). A co-transcript encoding a soymovirus polyprotein with coat protein (CP), AP, RT, RH1, and MP domains and a long chain acyl-CoA synthetase 1 domain was identified in alfalfa (*Medicago sativa*; Table 1 and Figure 1).

**TABLE 1:** List of co-transcripts containing caulimovirid and plant sequences identified in this work.

Source	ID	Species	Family	Group	Number of co-transcripts	Host domain/function	Virus domain
OneKp	EZZT	*Passiflora edulis*	*Passifloraceae*	Rosids	1	V-type ATP synthase subunit I	AP
TSA, NCBI	GGXZ02	*Platanus x hispanica*	*Platanaceae*	Basal Eudicots	2	Archaeal histone H3/H4	AP
OneKp	BDJQ	*Zingiber officinale*	*Zingiberaceae*	Commelinids	1	SH3 domain protein	AP,RT
OneKp	HDSY	*Aerva persica*	*Amaranthaceae*	Core Eudicots	1	Laminin G domain	CP,AP,RT,RH
OneKp	TOKV	*Aphanopetalum resinosum*	*Aphanopetalaceae*	Core Eudicots	1	glycosyltransferase family 1 and related proteins with GTB topology	MP
OneKp	RKGT	*Eschscholzia californica*	*Papaveraceae*	Basal Eudicots	4	GTP-binding nuclear protein Ran	MP
OneKp	GTUO	*Huperzia selago*	*Huperziaceae*	Lycophytes	1	light-harvesting complex chlorophyll-a/b protein of photosystem I (Lhca)	MP
OneKp	XLIQ	*Iodes vitiginea*	*Icacinales*	Asterids	1	light-harvesting complex chlorophyll-a/b protein of photosystem I (Lhca)	MP
OneKp	TJES	*Spergularia media*	*Caryophyllaceae*	Core Eudicots	1	PPR repeat family	MP
OneKp	XSSD	*Amaranthus hybridus*	*Amaranthaceae*	Core Eudicots	2	molybdopterin-synthase adenylyltransferase MoeB	MP
OneKp	YUOM	*Toxicodendron radicans*	*Anacardiaceae*	Rosids	1	Polysaccharide Lyase Family 6	MP
OneKp	IPPG	*Heliotropium filiforme*	*Boraginaceae*	Asterids	3	Aldolase/RraA	MP
OneKp	SCEB	*Podocarpus coriaceus*	*Podocarpaceae*	Conifers	1	OPT oligopeptide transporter protein	MP
OneKp	PVGM	*Oncotheca balansae*	*Oncothecaceae*	Asterids	1	Ubiquitin-conjugating enzyme E2, catalytic (UBCc) domain.	MP
TSA, NCBI	GFVC01	*Jatropha curcas*	*Euphorbiaceae*	Rosids	1	Heat shock protein 70 (HSP70)	MP
OneKp	PUCW	*Agastache rugosa*	*Lamiaceae*	Asterids	2	Molecular chaperone IbpA, HSP20 family	MP
OneKp	WHNV	*Clinopodium serpyllifolium*	*Lamiaceae*	Asterids	2	Molecular chaperone IbpA, HSP20 family	MP
TSA, NCBI	GFRR01	*Ocimum tenuiflorum*	*Lamiaceae*	Asterids	1	Molecular chaperone IbpA, HSP20 family	MP
TSA, NCBI	GFBP01	*Juncus effusus*	*Juncaceae*	Commelinids	1	Molecular chaperone IbpA, HSP20 family	MP
TSA, NCBI	GALV01	*Gossypium hirsutum*	*Malvaceae*	Rosids	2	Molecular chaperone IbpA, HSP20 family	MP
TSA, NCBI	IADW01	*Orobanche minor*	*Orobanchaceae*	Asterids	5	Molecular chaperone IbpA, HSP20 family	MP
TSA, NCBI	GDKT01	*Vigna unguiculata*	*Fabaceae*	Rosids	1	LRR kinase	MP
TSA, NCBI	GDIZ01	*Silene conica*	*Caryophyllaceae*	Core Eudicots	1	Acyl transferase	MP
TSA, NCBI	GDJH01	*Silene conica*	*Caryophyllaceae*	Core Eudicots	1	acyl transferase	MP
TSA, NCBI	GCZN01	*Abies pinsapo*	*Pinaceae*	Conifers	1	Phage-related minor tail protein	MP
TSA, NCBI	GFLL01	*Pinus albicaulis*	*Pinaceae*	Conifers	2	enoyl_reductase_like	MP
TSA, NCBI	GGKA01	*Medicago sativa*	*Fabaceae*	Rosids	3	Long-chain acyl-CoA synthetase	MP,RT,RH,CP
OneKp	TPEM	*Platyspermation crassifolium*	*Escalloniaceae*	Asterids	1	Major intrinsic protein (MIP) superfamily.	RH
OneKp	WPHN	*Idiospermum australiense*	*Calycanthaceae*	Magnoliids	1	Peroxiredoxin (PRX) family,	RH
OneKp	YYPE	*Austrocedrus chilensis*	*Cupressaceae*	Conifers	1	Seed maturation protein.	RH
TSA, NCBI	GDIY01	*Silene conica*	*Caryophyllaceae*	Core Eudicots	3	RING-finger-containing ubiquitin ligase	RH
TSA, NCBI	GDQW01	*Panax ginseng*	*Apiineae*	Asterids	6	Cyclic nucleotide-binding domain.	RH
OneKp	LWCK	*Lycium barbarum*	*Solanaceae*	Asterids	1	molecular chaperone DnaK	RT
OneKp	JOPH	*Carapichea ipecacuanha*	*Rubiaceae*	Asterids	1	Photosystem I psaA/psaB protein	RT
OneKp	ZSGF	*Carapichea ipecacuanha*	*Rubiaceae*	Asterids	1	Photosystem I psaA/psaB protein	RT
OneKp	UFJN	*Diplazium wichurae*	*Athyriaceae*	Ferns	1	C-terminal domain of rhamnogalacturonan lyase	RT
OneKp	TJQY	*Kerria japonica*	*Rosaceae*	Rosids	1	chromosome segregation protein SMC	RT
OneKp	SLYR	*Cladrastis kentukea*	*Fabaceae*	Rosids	1	Sel1-like repeats	RT
OneKp	LQJY	*Solanum virginianum*	*Solanaceae*	Asterids	1	Tetratricopeptide repeat	RT
OneKp	QSNJ	*Taiwania cryptomerioides*	*Cupressaceae*	Conifers	1	core domain of the SPFH (stomatin, prohibitin, flotillin, and HflK/C) superfamily	RT
TSA, NCBI	GFZE01	*Catharanthus roseus*	*Apocynaceae*	Asterids	2	LRR Kinase protein	RT
TSA, NCBI	GBHJ01	*Withania somnifera*	*Solanaceae*	Asterids	3	GTPase domain	RT
TSA, NCBI	GGDV01	*Croton tiglium*	*Euphorbiaceae*	Rosids	5	leucine-rich repeat receptor-like protein kinase	RT
OneKp	KXSK	*Agave tequilana*	*Agavaceae*	Monocots	1	Trimeric dUTP diphosphatases	RT,RH
OneKp	UQCB	*Portulaca molokiniensis*	*Portulacaceae*	Core Eudicots	1	Cupredoxin superfamily	RT,RH
OneKp	UQCB	*Portulaca molokiniensis*	*Portulacaceae*	Core Eudicots	1	Largest subunit of RNA polymerase (RNAP), C-terminal domain	RT, RH
OneKp	KEGA	*Glycine soja*	*Fabaceae*	Rosids	1	*P. syringae* resistance	RT,RH
TSA, NCBI	GAYS01	*Solanum incanum*	*Solanaceae*	Asterids	2	Late blight resistance gene R1	RT,RH
TSA, NCBI	GAYR01	*Solanum melongena*	*Solanaceae*	Asterids	5	Late blight resistance gene R1	RT,RH
TSA, NCBI	GBEF01	*Solanum melongena*	*Solanaceae*	Asterids	2	Late blight resistance gene R1	RT,RH

Co-transcripts including caulimovirid RT-RH sequences and *R1* late blight resistance gene sequences were identified in brinjal eggplant (*S. melongena*) and in closely related species from the Eggplant clade (*S. incanum*; Table 1 and Figure 1). Similar co-transcripts were identified in the wild progenitor of the eggplant, *S. insanum*, from transcriptomes of the SRA database (Page et al., 2019). We selected these co-transcripts for further investigation because genomic and transcriptomic resources are available for their plant host species and because identical co-transcripts were identified in the transcriptomes of these three closely related *Solanum* species (Figure 1).

**FIGURE 2:**
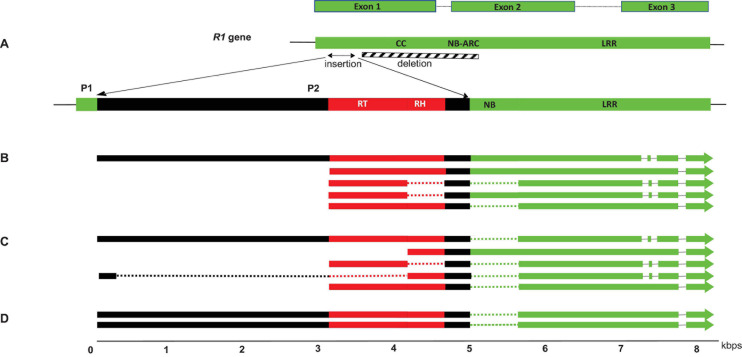
Structure of the VR1 locus of *S. melongena* **(A)** and co-transcripts identified in *S. incanum* **(B)**, *S. melongena* **(C)** and *S. insanum* **(D)**. Viral and host sequences are shown in red and green, respectively. Spliced sequences in the co-transcripts are represented by dotted lines and the deletion at *S. melongena* VR1 locus is represented as a hatched box. The position of alternate promoters P1 and P2 are shown.

Co-transcripts identified in *S. melongena*, *S. incanum* and *S. insanum* contained caulimovirid reverse transcriptase (RT) and RNAseH1 (RH1) domain sequences, and *R1* NB-ARC and LRR domain sequences (Figure 2). They ranged in size between 4 and 8 kb. Insertion of viral sequences resulted in multiple disruptions in *R1*, making translation into functional proteins unlikely. Mapping co-transcripts on the reference genome of *S. melongena* revealed a single insertion locus for this species, in which a 2.4 kbp sequence in the *R1* NB-ARC domain was replaced by a 5.8 kbp caulimovirid sequence (Figure 2). We named this locus virus *R1* (V*R1*). It is located at positions 432,102–443,945 bp on contig 05585 of S. *melongena* chromosome 0.

### Analysis of VR1 Co-transcripts

RNA-seq datasets from one *S. incanum*, two *S. insanum* and ten *S. melongena* accessions were used for analyzing V*R1* co-transcripts. Transcripts of different lengths (4–8 kb) were identified for the V*R1* locus of these three species, suggesting that alternative splicing occurs (Figure 2). Further evidence for alternative splicing was gathered using Splice Aware Aligner (STAR) and AS Finder-based pipeline. Five co-transcript variants (isoforms) were identified in *S. melongena*, five in *S. incanum* and two in *S. insanum* (Figure 2). The structure of these variants suggests that intron skipping and intron retention occurs in *S. incanum* and *S. melongena* and shows that *R1* highly conserved intron 1 (Ballvora et al., 2002) was expressed in *S. melongena* and *S. incanum* but not in *S. insanum* (Figure 2). Transcripts expressed in all three species retained the second intron located in the *R1* LRR domain (Figure 2). Short (4 kb) and long (8 kb) co-transcripts were mapped to the same insertion loci, suggesting the existence of alternative viral promoters in the V*R1* locus. Therefore, a search was done for alternative promoters in the V*R1* locus of *S. aethiopicum* and *S. melongena*. Two putative promoters (P1 and P2) were identified, potentially driving the synthesis of long and short transcripts, respectively (Figure 2). The P1 promoter is located upstream of the transcription start site (TSS) of the long transcripts. It displays 91.7% sequence identity with the closest homolog of the *R1* promoter of *S. aethiopicum* and *S. melongena*. The P2 promoter was identified upstream of the TSS of short transcripts located within the viral insertion and is therefore of viral origin. The TSS and NDPP programs predicted a promoter with linear discriminant factor scores of 1.977 (threshold = 1.52), and 0.97 (cutoff = 0.8), respectively. PlantCARE identified CAAT box motifs and a TATA-box located at 699 and 639 bp upstream of the TSS, respectively.

The transcription level of each co-transcript was assessed in one *S. incanum*, two *S. insanum*, and ten *S. melongena* accessions using the RNA-seq pipeline Tuxedo (Trapnell et al., 2012). Reads were mapped on *S. melongena* scaffold Ch0.5855 (Barchi et al., 2019). Transcription levels were very similar for all analyzed cultivars, except for *S. melongena* accessions Mel-4 and Mel-5, which displayed higher expression levels (Supplementary Figure 1). They were also similar to the transcription level (60,127.13 FPKM) of a constitutively expressed gene (ORC-6). These data suggest that co-transcripts were expressed constitutively in all analyzed *S. melongena*, *S. incanum* and *S. insanum* accessions.

### Classification of the EVE at the VR1 Locus

Endogenous viral elements at the V*R1* locus of *S. incanum*, *S. insanum*, and *S. melongena* had 100% nucleotide identity in the RT/RH1 coding regions, suggesting they derive from the same ancestral virus, for which the name brinjal badnavirus A (BBVA) is proposed (Supplementary Table 3). When a BLASTX search of the NCBI non-redundant protein database was done, matches were obtained to a range of badnaviruses, with the highest scoring match being to blackberry virus F (Sequence ID: YP_009229919.1). A near complete consensus genome of BBVA was assembled from the EVEs located on contig Ch0_17757 of *S. melongena*. The genome organization of BBVA is similar to that of badnaviruses^6^, including an open reading frame (ORF) 3 with putative MP, CP, AP, RT, and RH1 coding regions.

The reconstructed sequence of BBVA was used to search for related sequences in the recently sequenced nuclear genomes of *S. melongena* and its African relative *S. aethiopicum* (Barchi et al., 2019; Song et al., 2019). An additional badnaviral EVE was identified in *S. melongena* chromosome 1 (91008101–91010200) which only had 76% nt identity to the aforementioned sequences of BBVA in the RT/RNase H region and hence could be regarded as representing a different ancestral badnavirus, for which we propose the name brinjal badnavirus B (BBVB). Yet another distinct badnaviral sequence was identified in *S. aethiopicum*, for which the name gilo badnavirus (GBV) is proposed. This name is derived from gilo, the vernacular name of *S. aethiopicum*.

**FIGURE 3:**
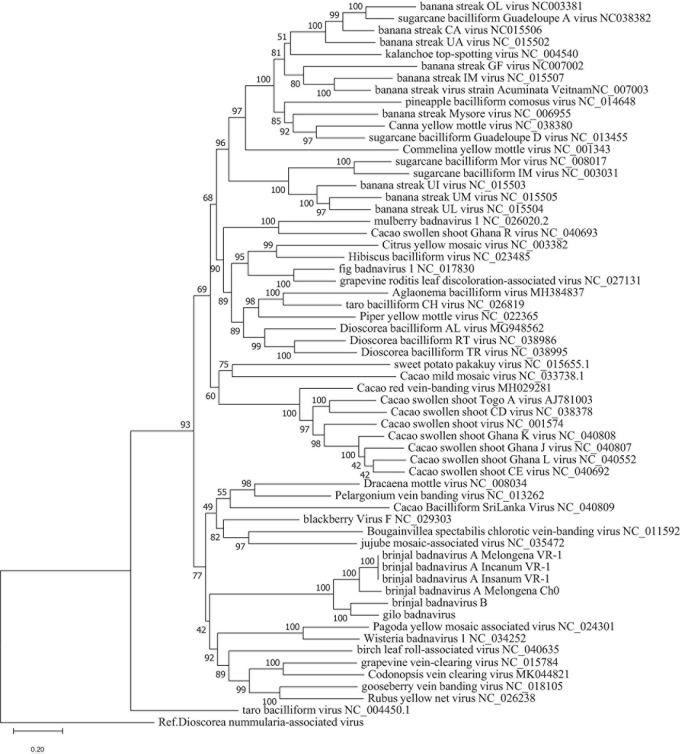
Phylogenetic tree showing placement of BBVA, BBVB, and GBV in genus *Badnavirus* using ML method. Analysis with 1000 bootstrap sets were performed on nucleotide sequences of the RT-RNase H domains corresponding to positions 5,599–6,882 in the genome of Commelina yellow mosaic virus (NC_001343), the type member of genus *Badnavirus*. Sequences of the proposed new species are shown in light red box.

To confirm classification, phylogenetic analyses were done using all EVEs that were found. The topologies of the trees obtained using maximum likelihood (Figure 3) and Bayesian methods (Supplementary Figure 2) were similar, with the EVEs included within a larger clade of eudicot-infecting badnaviruses originating mainly from the Old World. No evidence of recombination between BBVA, BBVB, GBV and other members of genus *Badnavirus* could be found following the analysis of badnavirus genome sequence alignments using the recombination detection program RDP4 (Martin et al., 2015).

### Endogenization of BBVA

Searches for BBVA EVEs were extended to 11 additional *Solanum* species from the tomato clade (*S. chilense*, *S. galapagense, S. lycopersicum, S. pennelli, S. peruvianum*, and *S. pimpinellifolium*), the potato clade (*S. demissum, S. tubersoum*, and *S. verrucosum*) and the Eggplant clade (*S. incanum*, *S. insanum*). For this, BlastN analyses were performed on complete genome sequences available for species in the tomato and potato clades using the assembled BBVA genome sequence as bait. Similar analyses were performed on transcriptomic datasets available for *S. incanum* and *S. insanum*, for which no genome sequence data is available. All screened *Solanum* species were devoid of BBVA insertions except *S. incanum* and *S. insanum* as mentioned above.

**FIGURE 4:**
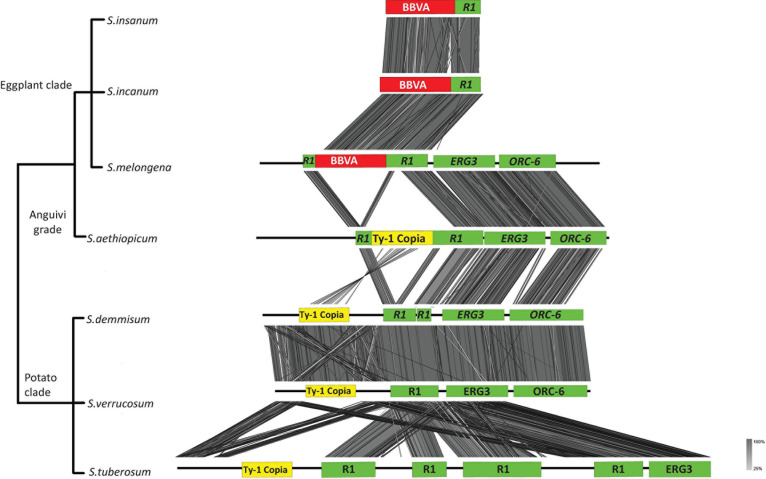
Syntenic relationships between S. *melongena* V*R1* locus and homologous genomic regions of related *Solanum* species. Viral sequences are shown in red and host sequences are shown in green. Ty-1 copia transposable elements are shown in yellow boxes. ERG3: sterol-C5(6)-desaturase; ORC-6: origin recognition complex 6.

*Solanum melongena* genomic sequences surrounding BBVA sequences at the V*R1* locus are flanked by an *R1* ortholog, sterol-C5(6)-desaturase (ERG3) (PFAM ID: PF01222) and ORC-6 (PFAM ID: PF05460) (Figure 4). Syntenic relationships were observed for BBVA, *R1* and ERG3 sequences between members of the Eggplant clade. Synteny was also observed for *R1*, ERG3 and ORC-6 between the genome of *S. melongena* and those of *S. aethiopicum*, *S. demissum*, *S. tuberosum*, and *S. verrucosum*, which are devoid of endogenous BBVA sequences. The V*R1* loci is very conserved between *S. incanum*, *S. insanum*, and *S. melongena* with nucleotide sequence identities of 98–99%.

**FIGURE 5:**
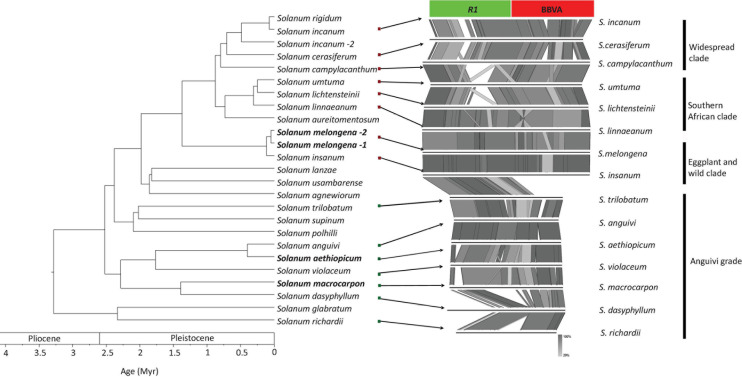
Phylogenetic relationships of *S. melongena* and related *Solanum* species along with syntenic relationships of the left border of the V*R1* locus. Left part of the figure reproduced from Aubriot et al., 2018).

PCR-based screenings were performed on *Solanum* species directly related to *S. melongena* (i.e., members of the Eggplant clade and Anguivi grade for which genome sequences are not available) using primer pair InlF2/InlR2, which allow the amplification of the entire V*R1* locus (Supplementary Table 4). Twenty-six accessions representing 17 species were screened (Supplementary Table 2). Amplification products of the expected size (6,866 bp) were obtained for 18 accessions representing seven species from the Eggplant clade (*S. incanum*, *S. insanum*, *S. linnaeanum*, *S. melongena*, *S. rigidum*, and *S. umtuma*) indicating the presence of endogenous BBVA sequences in the genomes of these species (Supplementary Figure 3B). Smaller amplicons (∼6,500 bp) were obtained for *S. campylacanthum* and *S. lichtensteinii*, whereas two amplicons of 4 and 1.5 kbp were obtained for *S. cerasiferum*. Attempts to clone these PCR products were unsuccessful due to their large sizes. Amplification products of 1.5 kbp were also obtained for species from the Anguivi grade (*S. aethiopicum*, *S. anguivi*, *S. dasyphyllum*, *S. macrocarpon, S. richardii*, *S. trilobatum*, and *S. violaceum* [see Aubriot et al. (2018) for details on these species] using the same primer pair (Supplementary Figure 3C). These amplification products were cloned and sequenced. Sequence analyses showed that they were devoid of BBVA sequences. These sequences were used in the synteny analyses illustrated in Figure 5.

These results suggest that endogenization of BBVA sequences could be a derived trait for the Eggplant clade and that a hypothetical ancestral lineage of the Eggplant might already have an endogenized BBVA sequence: BBVA insertion would have followed the divergence of the lineage formed by the eggplant and its closely related wild species (the Eggplant clade) from the other *Solanum* lineages (including the closely related Anguivi grade that accounts for *S. aethiopicum*).

Additional PCRs were performed using primer pairs InlF2/R1U and InrF2/R1D (Supplementary Table 4 and Figure 5) to amplify the left and right borders of the V*R1* locus, respectively, from the six species for which the complete VR1 locus was amplified (*S. incanum*, *S. insanum*, *S. linnaeanum*, *S. melongena*, *S. rigidum*, and *S. umtuma*). Amplicons corresponding to the right borders could not be obtained, despite several attempts and the design and use of alternative primers to primer R1D in the viral part of the right border. These alternative primers and primer R1D target the highly conserved NB-ARC domain of *R1* genes and other NB-LRR genes that are scattered across the genome of *S. melongena* (Barchi et al., 2019). This might explain why specific PCR products could not be obtained, thus preventing the sequencing of the right border. In contrast, amplicons of the expected size (∼1,545 bp) corresponding to the left border were obtained for all six species (Supplementary Figure 3D). These amplicons, which include the junction between the *R1* gene and BBVA sequences, were cloned and sequenced. Sequence comparisons showed that they display 84.4–99.1% nucleotide identity to each other (Supplementary Data File 1).

Analysis of the synteny between genomic regions corresponding to the left border of *S. melongena* V*R1* locus and comprising the *R1*/BBVA junction was carried out for all *Solanum* species for which this region could be amplified, cloned and sequenced (Figure 5). It showed that synteny between 8 species belonging to three of the four subclades within the Eggplant clade (widespread clade: *S. campylacanthum*, *S. cerasiferum* and *S. incanum*; Southern African clade: *S. lichtensteinii*, *S. linnaeanum*, and *S. umtuma;* eggplant and wild relative: *S. insanum* and *S. melongena*) and five species from the Anguivi grade (*S. aethiopicum*, *S. anguivi*, *S. dasyphyllum*, *S. macrocarpon*, *S. richardii*, *S. trilobatum*, and *S. violaceum*) is coherent with the phylogenetic framework proposed by Aubriot et al. (2018) for the Eggplant clade.

## Discussion

The contribution of EVEs to the biology of their hosts through structural and/or functional modifications of their genomes is well documented in mammals (Feschotte and Gilbert, 2012) but it is more limited in plants. However, the widespread presence of caulimovirid EVEs in plant genomes (Geering et al., 2014; Diop et al., 2018) and their conservation throughout plant evolution raise questions about their contribution to plant biology. In this study, we performed a comprehensive search for co-transcripts including plant and caulimovirid sequences in publicly available databases. We identified 50 such co-transcripts in 45 plant species belonging to 31 distinct families of vascular plants, providing evidence that fused ORFs including caulimovirid sequences are present in plant genomes.

Three of these co-transcripts were characterized in brinjal eggplant (*Solanum melongena*), and its close relatives *S. insanum* and *S. incanum*, providing the first evidence of badnavirus infections in the *Solanaceae* since no extant badnavirus has yet been reported for this economically important family. According to our analyses, BBVA inserted into gene *R1*, a NBS-LRR gene involved in resistance against *Phytophtora infestans*. *R1* belongs to the multigenic leucine zipper/NBS/LRR class of plant resistance genes and confers resistance against *P. infestans* in potato (Ballvora et al., 2002; Kuang et al., 2005) and other *Solanum* species including *S. melongena*. *R1* is used for breeding late blight-resistant crop cultivars (Faino et al., 2010), although *R1*-mediated resistance introgressed through breeding can be rapidly overcome by new strains of *P. infestans* (Fry and Goodwin, 1997). *R1* genes typically encode a 1,293 amino-acid residue polypeptide containing a coiled coil (CC) domain, a nucleotide-binding ARC domain (NB-ARC) and a leucine rich repeats (LRR) domain (Figure 2). Given the divergence dates and biogeographical reconstructions obtained by Aubriot et al. (2018), we suggest that BBVA endogenization has probably occurred once, in northern Africa or the Middle-East region during the last ∼ 3 Myr. *P. infestans* is considered to have originated from Mexico (Grünwald and Flier, 2005; Goss et al., 2014) and therefore there would not have been encounters between this pathogen and eggplant until relatively recently, certainly not before the discovery of the New World by Christopher Colombus in 1492. Hence, at the time of integration of BBVA, the plant population would not have been under selection pressure from *P. infestans* and gene *R1* may have been redundant in function. Endogenization of BBVA may have contributed to diversification of this gene family, and provided some positive benefit to the plant, particularly as this endogenous viral element has been retained through several plant speciation events.

Although splicing was reported before in the expression of rice tungro bacilliform virus (RTBV), a member of family *Caulimoviridae* (Fütterer et al., 1994), the impact of EVEs on alternate splicing is not yet documented in plants. Here, we provide evidence that the insertion of BBVA has disrupted *R1* and caused intron splicing. Transcriptomic analyses showed that several spliced transcripts of *S. melongena* and *S. incanum* display exon skipping and intron retention. Alternative splicing is known to be involved in the rearrangement of domains with preexisting functions into new protein composite architectures through exon shuffling, resulting in the acquisition of important new functions in eukaryotes hosts such as host-transposase fusion (HTF) genes (Cosby et al., 2021). Our work provides evidence that caulimovirid EVEs can promote alternative splicing in plants and have the potential to help forming fusion proteins with new functionalities. Premature adenylation was not observed in any co-transcript as all share a common 3′ end sequence. In contrast, two different 5′ ends were identified, suggesting the existence of alternative promoters. *In silico* promoter analysis suggested that a putative promoter (P1) from the *R1* gene might be driving the expression of long transcripts (8 kb) whereas a putative viral promoter (P2) identified within the BBVA insertion might be driving the expression of shorter transcripts (4–5 kb). Conserved caulimovirid *cis-*elements (TATA box and CAAT box) were identified in P2. Although co-option of viral promoters from EVEs was not reported before in plants, that of promoters from ERVs by animal genomes is known to contribute to the regulation of the expression of mammalian genes and to the transcription of non-coding genes (ncRNAs). Promoters acquired from ERV LTR regions also function as alternative and tissue-specific promoters (Faulkner et al., 2009; Beyer et al., 2011), whereas some retrotransposon internal coding sequences can serve as promoters.

Fast Unconstrained Bayesian AppRoximation (FUBAR, Murrell et al., 2013) analyses were performed on the RTs from BBVA, BBVB and GBV. Posterior probabilities ≧ 0.9, were registered for 13/40 sites, providing evidence of purifying selection (Supplementary Table 5). These results suggest that badnaviral RT sequences are conserved in brinjal eggplant and its wild relatives and could potentially be translated into functional proteins.

As evolution of viral lineages are spatiotemporally coupled with divergence and spread of host population, they can bring novel types of data to the traditional phylogenetic context. Hence, comparisons of shared BBVA insertion confirm *S. insanum* as the closest relatives of the eggplant and corroborate the current delimitation of the Eggplant clade. Current data suggest that BBVA EVEs in the genome of the Eggplant clade species might originate from a single endogenization event that would have happened between the late Pliocene and the early Pleistocene. This hypothesis now needs to be statistically tested by using a much more broadly sampled phylogenetic framework that will allow to confirm whether BBVA insertion happened several times in the giant genus *Solanum* or if it is a true synapomorphy of the Eggplant clade.

## Data Availability Statement

The original contributions presented in the study are included in the article/Supplementary Material, further inquiries can be directed to the corresponding author.

## Author Contributions

SS conceived the study, performed the analyses, and analyzed the data with help from FM, AG, and XA. VS, SS, and P-YT drafted the manuscript. All authors contributed to the final version.

## Conflict of Interest

The authors declare that the research was conducted in the absence of any commercial or financial relationships that could be construed as a potential conflict of interest.

## Publisher’s Note

All claims expressed in this article are solely those of the authors and do not necessarily represent those of their affiliated organizations, or those of the publisher, the editors and the reviewers. Any product that may be evaluated in this article, or claim that may be made by its manufacturer, is not guaranteed or endorsed by the publisher.

## Footnotes

1 http://www.onekp.com/

2 https://www.ncbi.nlm.nih.gov/Structure/cdd/wrpsb.cgi

3 https://mjsull.github.io/Easyfig/

4 ftp://ftp.solgenomics.net/genomes/

5 https://www6.paca.inrae.fr/gafl_eng/Vegetable-Germplasm-Centre

6 https://talk.ictvonline.org/ictv-reports/ictv_online_report/reverse-transcribing-dna-and-rna-viruses/w/caulimoviridae/1361/genus-badnavirus
