# Supplementary material for: Insertion of Badnaviral DNA in the Late Blight Resistance Gene (R1a) of Brinjal Eggplant (Solanum melongena)
Source: Front Plant Sci. 2021 Jul 23;12:683681. doi: 10.3389/fpls.2021.683681 (PMC8346255; doi:10.3389/fpls.2021.683681)
Supplement: Supplementary file 1 [file Data_Sheet_1.zip › Supplementary Figure 2.pptx]

## Slide 1
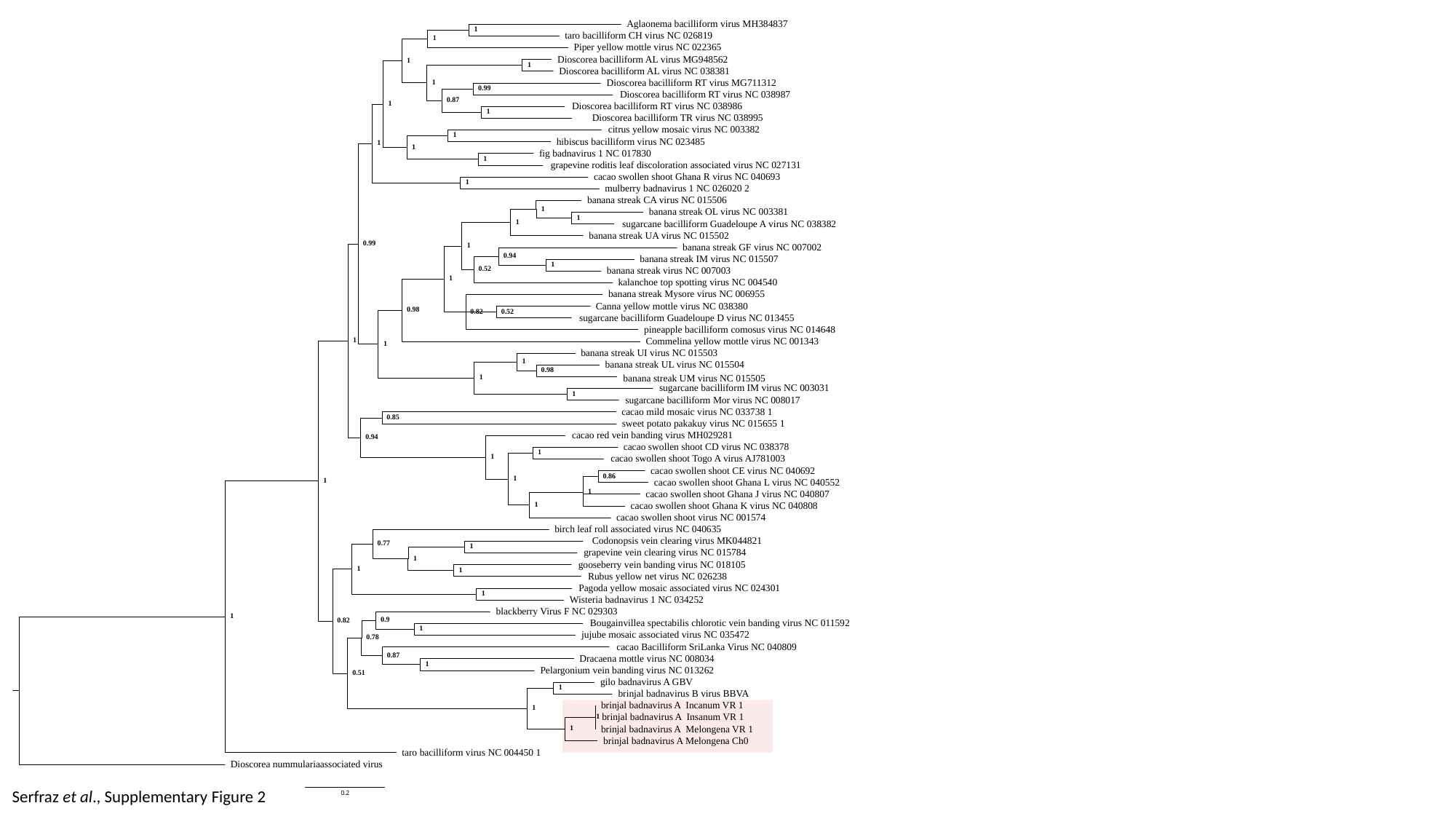

Aglaonema bacilliform virus MH384837
1
taro bacilliform CH virus NC 026819
banana streak IM virus NC 015507
0.2
1
Piper yellow mottle virus NC 022365
Dioscorea bacilliform AL virus MG948562
1
1
Dioscorea bacilliform AL virus NC 038381
Dioscorea bacilliform RT virus MG711312
1
0.99
Dioscorea bacilliform RT virus NC 038987
0.87
1
Dioscorea bacilliform RT virus NC 038986
1
Dioscorea bacilliform TR virus NC 038995
citrus yellow mosaic virus NC 003382
1
hibiscus bacilliform virus NC 023485
1
1
fig badnavirus 1 NC 017830
1
grapevine roditis leaf discoloration associated virus NC 027131
cacao swollen shoot Ghana R virus NC 040693
1
mulberry badnavirus 1 NC 026020 2
banana streak CA virus NC 015506
1
banana streak OL virus NC 003381
1
1
sugarcane bacilliform Guadeloupe A virus NC 038382
banana streak UA virus NC 015502
0.99
1
banana streak GF virus NC 007002
0.94
1
0.52
banana streak virus NC 007003
1
kalanchoe top spotting virus NC 004540
banana streak Mysore virus NC 006955
Canna yellow mottle virus NC 038380
0.98
0.82
0.52
sugarcane bacilliform Guadeloupe D virus NC 013455
pineapple bacilliform comosus virus NC 014648
Commelina yellow mottle virus NC 001343
1
1
banana streak UI virus NC 015503
1
banana streak UL virus NC 015504
0.98
banana streak UM virus NC 015505
1
sugarcane bacilliform IM virus NC 003031
1
sugarcane bacilliform Mor virus NC 008017
cacao mild mosaic virus NC 033738 1
0.85
sweet potato pakakuy virus NC 015655 1
cacao red vein banding virus MH029281
0.94
cacao swollen shoot CD virus NC 038378
1
1
cacao swollen shoot Togo A virus AJ781003
cacao swollen shoot CE virus NC 040692
0.86
1
1
cacao swollen shoot Ghana L virus NC 040552
1
cacao swollen shoot Ghana J virus NC 040807
cacao swollen shoot Ghana K virus NC 040808
1
cacao swollen shoot virus NC 001574
birch leaf roll associated virus NC 040635
Codonopsis vein clearing virus MK044821
0.77
1
grapevine vein clearing virus NC 015784
1
gooseberry vein banding virus NC 018105
1
1
Rubus yellow net virus NC 026238
Pagoda yellow mosaic associated virus NC 024301
1
Wisteria badnavirus 1 NC 034252
blackberry Virus F NC 029303
1
0.9
0.82
Bougainvillea spectabilis chlorotic vein banding virus NC 011592
1
jujube mosaic associated virus NC 035472
0.78
cacao Bacilliform SriLanka Virus NC 040809
0.87
Dracaena mottle virus NC 008034
1
Pelargonium vein banding virus NC 013262
0.51
gilo badnavirus A GBV
1
brinjal badnavirus B virus BBVA
brinjal badnavirus A Incanum VR 1
1
brinjal badnavirus A Insanum VR 1
1
brinjal badnavirus A Melongena VR 1
1
brinjal badnavirus A Melongena Ch0
taro bacilliform virus NC 004450 1
Dioscorea nummulariaassociated virus
Serfraz et al., Supplementary Figure 2
