# Supplementary material for: Insertion of Badnaviral DNA in the Late Blight Resistance Gene (R1a) of Brinjal Eggplant (Solanum melongena)
Source: Front Plant Sci. 2021 Jul 23;12:683681. doi: 10.3389/fpls.2021.683681 (PMC8346255; doi:10.3389/fpls.2021.683681)
Supplement: Supplementary file 1 [file Data_Sheet_1.zip › Supplementary material.docx]

***Supplementary Material***

# Supplementary Data

## BBVA, BBVB and GBV nucleotide sequences reconstructed from EVEs

>BBVA_near complete_genome, 7057bp

TGGTATCAGAACCAACAATAGGAGATGACGATATCGAAGAAACAGCTGAGGTCGCCGGCG

CCACATGGGTAGATGATCGACAGGGAGTATATGGCACCAAATGATGAAGAAGAAGAAGAA

TACGCCATAGAAATACCCGGAGAAGAGGAGGAGTATGCCTATGTGGTCACTCAAGGTTGG

ATAGAAGCAACACGACGCATATACGAACAAGAAGCAACTCGAATAGAGGAGCAAAGGCGG

CGTGATGAGGAAAACCAACATGCCCAAGAAAAATATGAGGAAAAAAGAAAGGCAGCAATT

GAGGCACAAAGAAGGAAAGAGGATGTCCGCATAACAATGATGAGAAGGCATCCAAAGTAG

AGGAAATCCAACTGGGATTTCCACTACGATGGTCGTAAATACCAATACATATGTAACTAT

GATCCACCATCGTCATCAAGTATCCCCATTGAAACCATCACAGCTCAGGGATGGGGAGAA

GAGTTTGATGAACCTGAGCAAAGACCGGAGCAACCAGCCGTAGCAGTAATTCAGTCTGAA

GATGACCAATTCAGTCTCCAGGGATTGCTCGGATCCCATTGGATGCCAGGTAACTAACTA

CCCCATCCACATGTCAGCCTCCCGACCATCGCCCTAGTTGTTAGGAGATTGGGCTCTCTC

CCTGTCCAGTTGTCGTATCCTCTGGTTTGCACTATCAGTTCCAGTCTATTTCTGGCCGCT

TCAATCGTCATTAGTGTATCAGGGACAAAATACACTAATTGTGAACCCTGGGATAGGTCC

ACCTCTGCTATGACTGTTATTACTCTGTCATCGGTCCAGTTGGTTTATCGTAGAGCAACT

AGTGCCATCGTCCCGGCACCTCTCCTGTGTAATGTCTGGAGCCTGATCATTATTAGCCCG

ATGTGGACTAACGTGTATCCTTCCGCTCTAAGCCTTTCATAACTTTGAGGGTTTATGAGG

CTTATCTGCTCCTGTTGTTCGTCCACCAGCCGGATCTTCTATGCTTCGTCTTACCGGTTG

GATTTGCCAACTCTCATTTAGTAACTGCTCAGTGGAGTGCTGTTCTCCAGGGATTGCTCG

GATCCCATTGGATGCCAGGTAATTAACTACCCCATCCACATGGTATCTGAACGTTGTATG

GCTGGTATTTGTCAGCCTCCCGACCATCGCCCTAGTTGTTAGGAGATTGGGCTTTCTCCT

TGTCCAGCTGTCGTATCCTCTGGTTTTGGATGAAAGGGTATCTGAAGGTGCGAAATGGTC

GTACCCTGTTACCCAGGATGAAAGGGTATCTGAAGGTGCGAATCCTAGTCAACGTAGTGT

AAGGGACTCAATTGAGTCGTGTCCTACTACGGAGACTGTAAGGGTGCCTGATGATACGAG

TCCTATCCAGGATAATGTATGGGACCATGAAGAGGAGCTAAGTAAGCAAGGCTATGTGGC

AGAAAAAGAGAACTGGGACATGGAATACCCAGTATTTGCTACAAAGGAGCATACAGCCAA

GATGGAGGACAGTGGGACGACATCAGCCATTAGCCCATATAGCAGACCCCCGGAGCCAAC

AATGGGGCAAGTAAACTACCCACCTGCAACAAGGAGGCCTGATACAAAGGTGGATGATAT

TAGGCAAAAAACAGAGGCCTACAGAAGAAGAGCATCCCGGGAACTGCCTACGGCTCAAGC

AAAATCTGGGGTAATATTAGTGCTCCCAGATGATTTCAGTAAGACCACAGATGTAATAGA

TAGATGGGAATCGATCACGAAGAATCACGTCAGCAGAATCACATGGTCAAGTAACCAAGA

GAAGGTGGATTACATAGAAAACCTAATGGGCGAAACTGAAAAGAAGACCTGGATGCAGTG

GCGGCTGGCATTCAGGAACAAGTATGATTCCTTGGTAGAAATTGCTGACGATACCCAGAA

TATCACATCACAAGTTAGGCAGATATTCATACTTGAAGATCCTGCACAGGGTTCAACGGA

ATTACAGGAACGTGCACTACAGGATCTGGAGAGACTTTCCTGTGAGAAAGTCAAGAATGT

ATGGAGCTACATGAGGGACTACAGGAGACTTGCAGCACTATCTGGGAGATCATACCTTCC

GGATACCTCAGACAAGTTCTTCAAGAAATTGCCACCAGCGATAGGGCCAACCATCGAGAA

AAAGTTCTTGGAAAAATACCCGGGGTACAACCCTGGGTGTAGAACCAAGATTGTTTTTTA

CATACCACTACCTCTCAGGAGTGTGTAAGGAAATGGCCATGCAAAGAGAGCTTAAGGATT

TATCCTTTTGCTCGGAAGTACCAATACCAGGCTACTATAGGGACAAGAGTGACAAGAAAT

ACGGGCTGAGAAAGTCCAAGTCCTATGCCGGTAAACCCCACAAAACTCACCTGAAGCTTG

TAAAGAATAAAGATGTCAAGCGTGGTTTTGTAAAGAAATGCAAGTGTTACTCCTGAGGAG

AAATTGGGCACATGCGACATGAATGCCCTAACAATACGGGAAACCTTGCAAGAGCTTCAA

TACTGAATGAGCTAGACATACCAGAGGGGTATGATGTCCTATCAGTAGAAGCGGACGAAT

CAGATAGAGATGACATATTCTCTATCTCAGAAGGAGAAGCAGGTGCAGTAATGCGAGAGA

CATTATTGCAGGAACTAGAAGGAGCATTCGTACTAATCGAGGAATGCCGGCACAACTGGA

GCTTTGATAAGGCGGCTTCATTGTTCAAATGTGAGCAATGTAAGCTTAACATAAACCAGA

GGCGGGTAGAATGCTCGGAATGCAAAGGTGAGATATGCGAGTTCTGCGCATGTCACCACC

ACAAAGTTAGAAAGCCGATGGAGATGAGGCTGACTACCTACATTGGGAAGGATGAGCTGA

TCAAGCAGCTACTTGAGTATATAGCCTTCCTACTACAGGAAAATGACAGGCTCAGGAAGT

CCACGGAGGAGGATTTCCAAGAGTTGGAAAGGGCCAGAAAGGGGAAAGCTATTGAAGGAG

AAGCTGGAACTAACGGAGATGACTACGCAACCCCTAAAGAAGAAGATGATGCAGCCGTAA

TGGAGGAATGGATATGAAATGTGGAAAAATCCATAACAGGACAGCAACCACAAGTAAGGC

AAGGAGCACTGAGTAGGCTCTACAACTTGTGCATCATAATAATGATCCCAGGAGTGCCTG

AATTCACTGTCAGAGGCATATTGGACACCGGTGCTACATAGTGCTGCATTAATGAGAATG

TGATACCAAAGGATGCAGTGGAGGAAACCGGGTATTCCATTGTGATTAACGGACTCAACT

CAAAACAGAGGTCCAAAAATAAATTAAAAAATGGAATGTGGGACATAGGAGGCAACAGGT

TCATAATCCCATTTACCTATAGTCTGCCTATGAACATAAGGGATGGGATCGACATACTAG

TTGGCTGCAATTTCATCAGAGCCAACGCGGGAGGCATGCGGATCGAAGGGAATGACGTCA

CCTTCTACAAAAATGTAGCAAGGATTGATACACGACCCAGAGCAGAGCATGCAAAGGCCT

TAGAAGTGGAAGGTCTAGAGCTAGCAGAGGCCCAAGAAGTCATGTACCTCAATTGGGGGA

GCCCAAGCCCAACTTTCAGGCAAAGGTTCCAAGCCATCATCAACAGGCTTAAGGGAATCG

GGATCGTAGGAGAAAGCCCATTACAGCATTGGGCCAGAAACAAAATCCGGTGTAAGTTGA

ATATCATTAACCCGGATACCACGATCCAGGATAGGCCTCTCAAGCACGTGACAGCCCAAA

TGGAGGAAGACTTTAACAAGCACATGCAGGCGCTATTGCGACTAGGGGTAATAAGGAAAT

CAAACAGTTGCCATAGAACAGTTGCGGTGTTGGTAAACTCTGAAACCAGCATCGATCCAG

CTACCGGAAAGGAGGTCAAGGGAAAGCAAAGAATGGTCTTTGACTACCGTGCGCTCAATG

ATAATACGCATAAAGACCAGTACTCTTTGCCAGGGATTGAAAGCCTGAAGCTCAGAATAG

GCAGATCAAAACTTTACTCGAAGTTTGATTTGAAGTCTGGCTTTCATCAGATTGCTATGG

ATGAAGAGTCAGTACCATGGACGACATTCCTGGTACCCGGGGGGCTCTATGAATGGCTTA

TCATGCCGTTCGAACTGAAAAATGCACCAGCAACATTTCAACGAAAGATGGACCACTGCT

TTGCGGGAATGGAGAAGTTCACGGCAGTATATATCGATGATATACTGGTGTTCTCTGACA

CTGAGGAGGAGCATGCTAAGCACCTTATGCAAGTGCTTGAAGTATGCGAGAAGAATGGGC

TCACCCTCACCGAAAACAAAATAAAGATCGCATGTTCGGAGATCGAGTTCTTAGGAGCCA

TCTTTAATGGCAGAAGGATGAAGCTGCAACCACACATAGTAAAAAAAGATCTGCGCAGTT

AAGGGAGAAGACATGCAGACGAAGAAAGGATTGCAGTCATGGCTTGGGATAGTAAACTAC

GCTAGGCCCCACATAAAGGACACAGGAAAGCTCCTTGGGCCACTCTATTCCAAAACCTCA

CCCCATGGTGATAAAAGGTTCAAGCCTTCTGACTGGGATTTGGTGAATAAAATCAAGGAA

TTAGTCAACAACCTACCGGACATGACTATTCCACCAAAGGAGGCCCATATTGTGCTTGAA

GTTGATGGATGTACGGAGGGATGTGGAGGAGTCTGCAAGTGGAAATGGAAGAAGGAAGAC

CCAAAAAATATGGAGCAGGTAAGTGCATATGCATCAGGAAAGTTTCCAACGGTAAAATCG

CCGGTGGATGCTGAAATCTATGCCTGCATGGAGACCATGCAGAGGCTTAAGATGTACTAC

CTGGACAAAAGAGAGATAACCCTAAGAACAGATTGCGAGGCAATTGTTCGGTTCTACAAT

AAAACGGCAACCAATAAGCCATCCAGGGTTAGGTGGCTTGGTTTCATGGACTATCTGACG

GGGACTGGTATGGACATACATATAGAGCACATAGACGGAAAAAGCAACCGGTTCGCGGAC

GAATTGTCCAGACTAACGACGGCTTCTGTTTTGCAGGAACAACACCAACAGGAGGAAGCC

ATGGTAATCCTTGCGGATGGAGAAGAGACATGTCACACATGTCATTAGCAGCCCAACACA

CGGCCCAAATTGAAGAAATGCTAAGCCCAAGACCTGTAGGACTTGGAACCTGGATCCAAA

TGGAGGACGAGGCCCAGAGACTGCTTGAAAAAGCCCAGCAAGAGGCCCAAAGGAGTCTGA

ACCAATTGGTTCGGATCGTTTCGACACGGAGGTACCATTATCAAAGAAGGGCAACTAGGA

ACAATGTGTATGGCGACCGCTTATCTCAGGCCATATGTAAGCAACAGGCAATTCAACGGA

TCCTCGAACAGCTAGAAAGAGCAATGAGAGGAGATTAGGTGGAAGCACGTGGTGGGACCA

TCTAAAGTACATACGTGTGCGACCCAAAATGCTTGGACGGTGTAATGGGTCATTTATTTA

TCTGTCAGTAGGGTAGTCCCCCTACTATGTTTGTTGGTTTGTAATAATTCCTGGCTAGAT

GCTTCTCGGTGAAGTGAGTGCCAGCTAGAGTTTTTGTCGGTTCCTGTCTAGAAAGGAATC

TTTAGCTTTTTACTTTAGAACGTGGGAAACATGTTAAGACCCACGCTGAATCAGAAGGAT

AAGAGGGAATCTGAATTATTGCGACGATGGGGCCCAATGAGCACCCGGCAATAACCCAAG

AATATCCTTCTATAAATAGGCAGTAGTGTGTTCCATTTAAACACAGATCAGAATACCTGA

GAATGTTTATTTGATAAAAACCTTTGTACCCCTAAATAGAAAGCAATAAAATCTTTCTTT

TCTTTCTTCCTTTTGAGCAACAACTAGTAGACTCTGGAAGACGATCCTTTTTCTTACTGC

TATCAGAGCTAATGGATCTAGAGGGGTAAAAGCCATTCTGGACTGATGGGAAGGTAAGGT

AAATACTCATCTTTTAAACTCTTAAGTGCTTATATTTATTTAAGTTTACTAATTTACCTT

AATTGTATTGTATGGTAATCTTAACCTGAGTGAATAGTCTCATTAAATCCTGAAGGGCTA

AGTAATAAGTATGTTATTAGTAAATGAAATCTGAGTTGACCAGTGCCTTGTTGTATTGTT

TACCTTTATTAATATCTTTCAGTTCCTAATGACCTATAGACCACAAGTAAGTCCTGTGGA

AAGGCAATTAAGTGGTTGCGGTTTGAAGGGATTGTTAGTTATTTAAGGTAACTATGCAAG

GACGCACGGGCCCTTAGAATATCATTACTAGTAACCTATGAAGTACTAGTTGGGTAAGGT

GAGATTCCAGGGGTAGCAACAACTATACAGTATAAAGATTGCAGAAAAGTTTACTTACAT

AAGTAATAAGTACTCTAGAATGTTTAAAGGATGATATGAACCTAAACCATACCCAGAGGG

CTAGAATTCTCACAAAATAGTAAAACCCACAAAAAGAGTGAAGCAGTGAATGAGTGACTG

GAAACGTACAGCCAAAATCCCAGCATATATTGAAGCTGTTAAAGCTACTGAAACAATAGA

AGAACCTGCTGTTGGTTTCTATAAACCATCAGATTCTAGATTCGCACAAAACGTGGTAAT

AATAAAACAAAATAATACCATAATCCAACTGTTAACCAAACTCACAGAAGAAGTAGCAGA

TCTCAAGGAGGACTTTGGTATACTCAAGGGAACTATAAGCAGAAAGCCCTTGGACAATGG

GAAAACTAAGGAAGATCTCAGTGAATATCTGGAGAATATCCAGAAGAAGCTAGACAACTT

TTACGTTGGTGATCCAAAACCATCAACAACAAAAGTCAAAAAATCGCCATTCTACGTCTT

CAAGGACCCCCAGCAAATTTACAAAGATGCAAAGAAGCAGGATACCTGACCTGGCAAGTG

TCAGAAGTTCGGAGGAGGAACAGACGCCGTCTCAGGAGGACCAAATCCGTAGCTACATAC

GTGGTGCAAGGATCAGGCATGAAGTGGGCAGAAGACT

>BBVB_Ch01_pol_sequence, 1422 bp

TTGGCTCATTGGTGCATTTATTCTCATAGCATCATAATTATTGTCATTTAGCCAATGATT

AAATGCAAAATAGATATCATAAATCCTGAATTCACGATCCAAGATAAGCCACTAAAACAT

GTCATGGCCCAGATGGAAGCGAACTTCAAAAGACATATAAAGGCACTACTAGAGCTGGGA

GTAATAAGGGAGTCTTCAAGCTGGCACAGAACAACATCAGTATTGGTAAATTCCAGAACT

AGTATCAATCCAGCTACTGGAAGAGAGGTGAAAGGAAAACAAAGAATGGTCTTTGACTAC

AGGGCAATAGATGACAACACCCATAAGGACCAATATTCTTTGCCTGGGATTGAAAGCTTG

AACTAAGAATTGGCAGGTCAAAGCTATACTCAAAGTTTGACTGAAAGTCTGGCTTTCACC

AAATCACAATGGAGGAAGAGTCCATACCATGGACGGTGTTCTTCGTACCTGGAGGAGGAC

TTTATGAATGGCTAGTAATGCCATTTGGTCTGAAGAATACACCAGCAGCATTTCAACGCA

AGATAGATCACTGCTTCAAAGGAATGGAGGATTTTACGATAGTATACATTGATAATATAC

TGGTATTATCAGATACTGAGGAGCAGCATGCCAAACACCTAGAGCATTTACGAAAGTTAT

ACGAGAAGGAAGGGCTCATACTCTCCGAAAATAAAATGAAAATTGCCTATAAAGAGAATG

AGTTCCTTAGAGTCGTCTTTAATGGGAGAAAAATGTGATTGCAACCAAACATAGTCAAAA

AGATTTGCGCAGTTAAGGGAGAAGACCTACAGACGAAGAAAGGGTTGCAGTCCTGGCTTG

GAATAGTGAATTACGCCAGGCCCCACATAAAGGAAATAGGGAAACTCCTAGGACCACTCT

ATTCCAAAACCTCACCCCATGGCGATAAAAGATTCAAGCCTTCTGACTGGGCACTGGTCA

ACCGCATCAAAGAGGCAGTCAACAACCTCCCAGATATGACGGTACCACCAAATGACGCCC

ATATCATGCTTGAAGTAGATGGATGCATTAAGGGATGGGAGAGTTTGCAAATCGAAGACA

AAGAAGGTAGACCCTATTTCAACAGAATAGGTATGTACATACGCAAGTGGCAAGTTCCCT

ACAGTCAAATCACCAGTTGATGCCAAGATCTATGCCTGCATGGAAACCATGTAGAGACTG

AAGATGTACTACCTAGACAAAAAGGAGATCACCCTATGGACTGATTGCGAGGTAATAGTT

TGGTTCTACAACAAAACTGCTACCAATAAGCCGTCAAGGGTGAGATGGCTTGGGTTCATG

GACTTCATCACCGGCTCTGGAGTATATGTTCACATCGAGCATATTGATGGGAAGAAGAAC

AAGCTGGCAGATGAGTTGTCCAGACTAATAATGGATTTTGTT

>GBV_scaffold897_cov62_pol_sequence, 1711 bp

AACAGGCGTGTACTCAACAAACTGTACAACCTTACAGTCACCCTGATGATCCCTGGAATA

GCTGAATTTAGTGTAAGAGTTATTCTAGATACTGGAGCTACCTCGTGCTGCATCAATCAA

GAAGTGATACCAAAAGGAGCAATAGAGGAAACGGGGTACTACATCACCATCAATGGGCTG

AATTCAAAACAACAATCAAAACATAAGCTCAAAGATGGAATGATGGACATCGGAGGCAGC

AGGTTCAGAATCCCGTTCACTTACAGCCTTCCGATGATAATCAGGGACGGGATAGACATG

TTCATCGGGTGTAACTTCATCCGAGCAGCTGCAGGAGGCATAAGGATTGAAGGGAATAAC

GTCACCTTCTACAAAAATGTGACGTCCATTGACACTAGGCCTCACATTGAGCATGCAAAG

GCATAGAGCTAGAAGGCCTGGAACTACTGGATGCCCAGGAAGCAGTCCTACTAAACTGGG

GAAGCCCAAGCCCAACATTCAGACAGAAGTTCCAGATCATAATAGATAGGCTAAAGGGGC

AGGGATCGTTGGAGAAAGCCCACTAAGGCATTGGGCCAGGAATAAAATCAAATGCAGGAT

TGATATTATAAATCCTGAAATAACGATCCAGGATAAGCCTATGAAACATGTCACGGCCCA

GATGGAAGCAGACTTCAGAAGACATGTTGAGGCATTAAGAGAACTTGGAGTAATCAGGGA

ATCTTCAAGTCGGCACAGGACAACGGCGGTACTAGTAAACTCCGGGACTAGTATCGACCC

TGCTACCGGAAGGGAGGTAAAAGGGAAGCAAAGAATGGTCTTTGACTATAGGGCATTAAA

TGACAACACCTATAAAGACCAATACTCTTTGTCAGGGATTGAAAGCCTGAAGTTGCGCAT

AGGAAGGTCAAAACTTTACTCAAAATTTGACCTCAAATCCGGCTTTCATCAAATCGCAAT

GGAGGAAGAGTCGATACCATGGACGGCGTTCTTGGTACCAGGAGGAGGACTCTACGAATG

GTTGGTAATGCCATTCGGCCTGAAAAATGCACCAGCAGCATTCCAGCGTAAAATGGACCA

TTGCTTCAAGGGAATGGAGGATTTTACGGCAGTATATATCGACGATATACTGGTGTTTTC

AGACACAGAAGAGCAGCATGCCAAACACCTGGAGCAGGTACTGAAGGTATGCGAGAAGGA

AGGACTAATACTCTCTGAAAATAAAATGAAGATCGCTTGTTCAGAGATCGAGTTCTTAGG

TGTCGTCTTCAATGGCAGAAAAATGAGGTTGCAGCCACACATAGTGAAGAAGATCTGCGC

AGTAAAAGGAGGAGATCTGCAGACAAAACGAGGGTTGCAATCCTGGCTGGGAATAGTAAA

TTATGCAAGGCCCCACATAAAGGACATAGGGAAACTCCTTGGGCCACTCTATTCCAAAAC

CTCACCCCATGGAGATAAAAGGTTCAAGCCTTTGGATTGGGCTTTGGTGAATCAGGTCAA

AGAGGTTGTCAATAACCTCCCAGATATGGCAATACCACCAAACAACGCCCATATTGTGCT

TGAAGTCGATGGATGCATGGAAGGTTGGGGAGGAGTCTGCAAATGGAAAAAGAATAAGGC

AGAAATTATCTGTTTCAATTTCTTGGGCCATAAAATCCTACCAGTCCCCGCCAATGCCGC

CGGACCACCTGGGCCGCTTGCTGGGCTTATC

## Nucleotide sequences of the left borders of V*R1* loci from spiny solanums

>*S.aethiopicum*_1

CAAACAATACGGTACAACTCAACTATAATTCACTTAAAGCTCTTTACAGAATGCTTTCTATATTGATTGTAGCATTACATTTAGACAATAGTGGAACATTTGTCGAGCTTTGACGATAGAATCTTCCTATTTTTAGTTATATTAGTTTTTGGTAGCGTGTATTGCATGTTAGTCCTTGTTATTGTTATAAAATTTCTATATACTTAAAAAAGTAAGTTTCAAAATAGAAAATGGTAAAATGAACAGCATTAATTTTATCGTTTACATTTTTAGCTAGAAATATAGTACCCAGCAATAGTTTAACTTTAAACATCAAATTTTAAATGAAATAATAATTTAGTCTTTTTGTTAACTGAGTGTAATGTGTTCTTTTCCCAGGATGTATTTGGACAAAGAATTGTCTGGGAATATTCTGGGATGTTGCAAGACATCCAATAGATTTCTTTCTGTGGGAGTTAAAATTCGTTGATTGTTTTCTCCATTTCTCACAGGAAATGCTAGAAATTTTCAAGAGGATGTGTGATACATTATCTAGCATTTAGATGATGGATTTGCATACTGGAAGGAGGTAATTTGGAAGAMTAAGCAAGAATTCAGATCTAGATACTCCTTTCCACTAGCAGCCAACAATATTAATATTCATAGCAACRAATTTGTGATGGAATTCATCGATGCTGWTGAATCACTAGTGAATTCGCSGCCGCCTGCACGTCGACCATATGGGACACCTCCCAGCGCGTTGGATGCATAGCTTGAGTATTCTATAGCGTCACCTAAGTAGCTTGGCGTAATCATGGTCATAGCTGCTTCCAGAGAGAAATTGTCATCCGCTCACACTTCCACACAACATACCAGCCGGAAGCATAGAGTGAAGAGCCTGGGGTGCCTAAGGAGTGAACTAACTCAAATGAGTTGCGGTGGCACTCACTGCCCGCTTTCCAGTCAAGAAAACCTGTCGTGCCAGCTGCATTAATGAATCCGCCCACCNCCCGGGGAAGAGTCTGTTTTGCGTATTGGNNCCCNCTTTCCNNNCCCTCCCTCAATGACTCCCATTCGTTNGGTCGGTTGGGGGGGCGGCGAAGTGGTATCCCCTTCACTCAAAGGCGGGTTAATTCGGNCTTTCCCNNNGA

>*S.umtuma*

CAAACAATACGGTACAACTCAACTATTATTCACTTAAAGCTCTCTACAGAATGCTTTCTATATTGATTGTAGCATTACATTTAGATAATAGTGGAACATTTGTCGAGCTTTGAGGGTAGGATCTTCCTATTTTTAATTATACTAATTTTTGGTAACGTGTGTTGCATGTCAGTCCTTTGTTATTGCTATAAAGGTTTCTATATACTTAAAAAAGTAAGTTTAAAATAAAAAAATGGGAAATGAACAGCATTAATTTTATCGTTTACATTTTTAGCTGGAAATATAGTACCCACCAATAGTTTAACTTTAAACAAAATTTTTACATGTAAAAATAATTTAACCTTTTTTGTTAATATAAAAATTATATTCACAAAAAAAGGGACTTATTTAATATTGCAAAATTTGAAGTGGCTTTTTTTCCATGGCTAAAAATAAGGTCAAGATTAAACATTTTTCATATAAATTAAAAATTGAAGGTACGTTTCCTTTTCATCCAAAAATATTCCCAGGAAGTAAGTCAACAGTGTAAACTCGTTATTGCATTCCAATGGTGTTTTTCCCAGGTTAATTCTTGTGTTACAAGGATATATTTTGAAAAAGAATTGTCTGATCTGAGAAATCGCGTTGTTTGCAGATTGCAAACACGAATATTCTGGGATGTTGCTGTTCACATCCAATTTTTACCCTCCACGGTACAAATCAATAATCAAGCTTTTAATTTTAACAATTTGAAATAATTAGCTTTCGAAAATGCAAAAAATATTTTTCCAAATAAATCTCTTATTTTTTTATTTTTAGGAGTTATTATTCTTAATTATTTCGGGTTGATAATATTAAAAACTCTTTAATTAATTTGTATAATTCATCGTGTAGTCAAATTAGTCAATCGAATTCAATTTAATTATTTTTAATTGGCTAAAATTACAATTGAATTGGTCAATTTTAAATCCCCACCTTGAACCCATTTTCAGCTTTAATAATGGACCAATTCTTGGCTCAAATAAAGTCAACCCAACCCACTTTTAATCCCAAACCCGGCCCACTTCCCTTTTATTTTTCAGCATAATATAATATATATGTATATATGTTGCGCGTGTCCTACACGCGCCCCCAATATTTCCTTTTTTTCTCCTCATTTCCTCTCACGCGTATGCACCCACTCTCCCCCTTTTCGTTCTTCTCCTTCACCTCAACAAACCAGGCGACAACCAGCAACAAACCCGACAATCCAAGAATCTCTCCAACGTTACGATAGGCAAATTCGTACATACGTGGCTTCTTCTTAGCCTCTATGATACGAATCCGTTGAGGGAATTTCCAAGTG

>*S. violaceum*_28

CAAACAATACGGTACAACTCAACTATAATTCACTTCGAGGTCGAGCTTTGAGGGTAGGAAATTCCCATTTTTAGTTATACTAATTTTTGGAAACGTGTGTTGCATGTTAGTCCTTTATTATTGTTATAAAATTTCTATATACTTAAAAAAGTAAGTTTCAAAATAGAAAATGGTACAATGAACAACATTAATTTTATCGTTTACATTTTTAGCTAGAAATATAGTACCCAACAATAGTTTAACTTTAAACATCAAATTTTACATGTAACAATAATTTAGTCTTTTTGTTAAAATTAAATGTATAGTCACATAAAATGTACTTATTTATATTACAAATTTCAAAAGTCTTTTTTCATGCTTAAATTATGTCTAGTTTAACACTTTCGTATAAATTAAGAGTAGAGTAGCATATAATTTACTTGCAATATTTGACTTGGTAGTTGTAATTGACTCAATTAAAATATTGACATAATACATATAATCGTCCTCTCTAACATGACACGGTATAAATCAATTGTCATGTAGAAGAGGATAAATTGAAGGATATGTTTACATATTATGCCAAAAATAGTAAGTCAACGGATTGTAATACTCTGCATTGTATCTCAATTGTTCTAAAACAGTTTAATTCTTCTGTTACAAGGATGTATTTCGACAAAGAATTATCTGATCTGAAAAATGGCGTTGTTGCAGATTGGAAACACGAATATACTGGGATGTTGCAAGATATCCAATAGATTTCTGTGGGAGTTAAAATTCGTTGATTGCTTTCTCCATTTGCAAAGCGTCACTTTTGCAAGTGAATGTGGTATGCTACATTTCTCACAGGAAATGCTAGAAATTTTCAAGAGGATGTGTGATACATTATCTAGCATCCCTGATGCTAATTTAGATGATGGATTTGCATACTGGAAGGAGGTAATTTGGAATACTAAGCAACAATTCAGATCAAGATACTCCTTTCCACTTGCAGCCAACAATATTAATATTCATAGCCCCGAATTTGTGATGGAATTCATCGATGCTGTTG

>*S. dasyphyllum*_5

CAAMCACTMCGGTACAACKCAMCTATAATTCRTTGCAGCTCTTTRCAKAATGCKTTCTAKATTGRKTGAGGATTACMTTTATACRATWBTGGAACATTTGTCGAGCTTTGACSATAGAATCTTCCTATTTTTASWTMTATTARTTTTTGGWARCGTGTATTGCAYGTTAGTCCTTGTTATTGTTATAAAATTTCTATATACTTAAAAAAGTAAGTTTCAAAATAGAAAATGGTAAAATGAACAGCATTAATTTTATCGTTTACATTTTTAGCTAGAAATATAGTACCCAGCAATAGTTTAACTTTAAACATCAAATTTTAAATGAAATAATAATTTAGTCTTTTTGTTAACTGAGTGTAATGTGTTCTTTTCCCAGGATGTATTTGGACAAAGAATTGTCTGGGAATATTCTGGGATGTTGCAAGACATCCAATAGATTTCTTTCTGTGGGAGTTAAAATTCGTTGATTGTTTTCTCCATTTCTCACAGGAAAYGCTAGAAATTTTCAAGMGGATGTGTGATRCATTMTMTAGCATTTAGATGATGGATTWGCATRMTGGAAGGAGGTAATTTGGAAGAATAAGCAAGAATTCAGATCTAGATACTCCTTTCCACTAGCAGCCAACAATATTAATATTCATAGCAACAAATTTGTGATGGAATTCATCGATGCTGTTG

>*S. macrocarpon*_13

CAAACAATACGGTACAACTCAACTATAGTTCACTTAAAGCTCTTTATAGAATGCTTTTTATATTGATTGTAGCATTACATTTAGACAATAGTGGAACATTTGTCGAGTTTTGAGGTAGGATCTTCCTATATTGCATGTTAGTCCTTTGTTATTGTTATAAAATTTCTATATACTTAAAAAAGTACGTTTCAAAATAGAAAATGGTAAAATGAACAGCATTAATTTTATCGTTTACATTTTTAGCTAGAAATATAGTACCCAGCAACAGTTTAACTTTAAACATCAAATTTTACATGTAATAATAATTTAGTCTTTTTGTTAAAATTAAATTTATAGTCACATAAAATGTACTTATGTATATTACAAATTTGAAGAGTCTTTTTTCATGCTTAAATTATGTGTAGTTTAACACTTTCATATAAATTAAGAGTAGAGTAGCATATAATGTACTTGCAATAGTTGAGTTGGTAGTTGTAATTGACTCACATAATACATATAATCGTTCTCTCTAACATGGCACGGTATAAATCAATTGTCATGTAGATGAGGATACTTCTTAAACTTTATCCAAAATTGAAGGATACGTTTACGTATTATGCCAAAAATAGTAAGTCAACTGAGTGTAATACTCTGTATTGTATCTCAATTGTTCTTTTCCCAGTTTAAGTCTTGTGTTACAAGGATGTATTTCGACAAAGAATTGTCTTATTTGAAAAATCGTGTTGTTCTCAGATTGCAAACAGGAATATTCTGGAATGTTGCAAGACATCCAATAGATTTCTTTAAGTGGGGGTTAAAATTCGTTGAGTGTTTTCTCCATTTGCAAGTGAATGTGGTATGCTACATTTCTCACAGGAAATGCTAGAAATTTTCAAGAGGATGTGTGATACATTATCTAGCATCTCTGATGCTAAGTTAGATGATGGATTTGCATACTGGAGAAGGTAATTTGGAATACTAAGCAAGAATTCAGATCTAGATACTCCTTTGACAACAATATTAATATGCATAGCCCCGAATTTGTGATGGAATTCATCGATGCTGTTGA

>*S. trilobatum*_26

CAAACAATACGGTACAACTCAACTATAATTCACTTAAAGCTCTTTACAGAATACTTTTTATATAGATTGTAGCATTACATTTAGACGATAGCGGAACATTTGTCTAGCTTTGAGGATAGAATCTTCGTATTTTTAATTATATTAATTTTCGGTAACGTGTATTACGTGTTAGTCCTTCGTTATTGTTATAAAATTTCTATATACTTAAAAAAGTAAGTTTCAAAATAGAAAATGGTAACATGAACAGCATTAATTTTATCGTTTACATTTTTTGCTAGAAATATAGTACCCAACAATAGTTTAACTTTAAACATCAAATTTTACATGTAATAATAATTTAGTCTTTTTGTTAAAATTAAATTTATAGTCACATAAAATGTACTTATTTATATTACAAATTTGAAGTGTCTTTTTTCATGCTTAAATTATGTGTAGTTTAACACTTTCATATAAATTAAGAGTAGATACTTGCAATAGTTGACTTGGCAATTGTAATTGACTCTATTAAAATAATGACATAATACATATAATCATCCTCTCTAACATGGCACGGTATAAATCAATTGTCATGTAGAGAGGATACTTGTTAAACTTTATCCAAAATTGAAGGATATTATGCCAAAAATAGTAAGTCAACTGAGTGTAATACTCTGTATTGTATCTCAATTGTTCTTTTCCCAGTTTAAGTCTTGTGTTACAAGGATGTATTTCGACAAAGAATTGTGTGATCTGACAAATCGCAGATTGCAAACAGGAATATTCTGGGATGTTGCAAGACATCCAATAGATTTCTTTCTGTGGGAGTTAAAATTCGATGATTGTTTTCTCCATTTGCAAGTGAATGTGGTATGCTACATTTCTCACAGGAAATGCTAGAAATTTTCAAGAGGACGCATCCCTGATGCTAATTTAGATGATGGATTTGCATACTGGAAGGAGGTAATTTGGAATACTAAGCAAGAATTCAGATCTAGGTACTCCTTTCCACTTGCAGCATTAATATTCATAGCCCCGAATTTGTGATGGAATTCATCGATGCTGTTG

>*S. anguivi*_2

CAAACAATACGGTACAACTCAACTATAATTCACTTAAAGCTCTTTATAGAATGCTTTCTATATTGATTTTAGCATTACATTTAGACAATAGTGGAACATTTGTTGAGCTTTGAGGATAGAATCTTCCTATTTTTAGTTATATTAATTTTTGGTAACGTGTATTGCATGTTAGTCCTTGTTAGTGTTATAAAATTTCTATATACTTAAAAAAGTAAGTTTCAAAATAGAAAATGGTAAAATGAACAGCATTAATTTTATCGTTTACATTTTTAGCTAGAAATATAGTACCCAGCAATAGTTTAACTTTAAACATCAAATTTTACATGTAATAATAATTTAGTCTTTTTGTTAAAATTAAATTTATAGTCACATAAAATATACTTATTTATATTACAAATTTGAAGTGTCTTTTTTCATGCTTAAATTATGTCTAGTTTAACACTTTCATATAAATTAAGAGTAGATACTTGCAATAGTTGACTTGGTAGTTGTAATTGACTCAATATATAATCGTCCTCTCTAACATGGCACGGTATAAATTAATGGTCATGTAGAAGAGGATACTTGTTAAACTTTATCCAAAATTATGCCAAAAATAGTAAGTCAACTGAGTGTAATGTATCTCAATTGTTCTTTTCCCAGGATGTATTTCGACAAAGAATTGTGTGATCTGAAAAATCGCACAGGAATATTCTGGGACATCCAATAGATTTCTTTCTGTGGGAGTTAAAATTCGTTGATTGTTTTCTCCATTTGCAAGTGAATGTGGTATGGAAATTTGCAAGAGGATGTGTGATACATTATCTAGCATCCCTGATGCTAATTTAGATGATGGATTTGCATACTGGAAGGAGGTAATTTGGAAGACTAAGCAAGAATTCAGATCTAGATACTCCTTTCCACTTGCAGCCAACAATATTAATATTCATAGCCCCGAATTTGTGATGGAATTCATCGATGCTGTTG

>*S. richardii*_25

CAAACAATACGGTACAACTCAACTATAATTCACTTAAAGCTCTTTACAGAATGCTTTCTATATTGATTGTAGCATTACATTTAGACAATAGTGGAACATTTGTCGAGCTTTGACGATAAAATCTTCCTATTTTTAGTTATATTAATTTTTGGTAACGTGTATTGCATGTTAGTCCTTGTTATTGTTATAAAATTTCTATATACTTAAAAAAGTAAGTTTCAAAATAGAAAATGGTAAAATGAACAGCATTAATTTTATCGTTTACATTTTTAGCTAGAAATATAGTACCCAGCAATAGTTTAACTTTAAACATCAAATTTTAAATGAAATAATAATTTAGTCTTTTTGTTAAAATAAAATTTATAGTCACATAAAATGTACTTATTTATATTACAAATTTGAAGTGTCTTTTTTCATGCTTAAATTATGTCTAGTTTAACACTTTGATAAAAATTAAGAGTAGATACTTGCAATAGTTGACTTGGTAGTTGTAATTGAGTCAATGACATAATACATATAATCGTCCTCTCTAACATGGCACGGTATAAATCAATGGTTACTTGTTAAACTTTATCCAAAATTGAAGAATACGTAAGTCAACTGAGTGTAATGTGTTCTTTTCCCAGGATGTATTTGGACAAAGAATTGTCTGGGAATATTCTGGGATGTTGCAAGACATCCAATAGATTTCTTTCTGTGGGAGTTAAAATTCGTTGATTGTTTTCTCCATTTCTCACAGGAAATGCTAGAAATTTTCAAGAGGATGTGTGATACATTATCTAGCATTTAGATGATGGATTTGCATACTGGAAGGAGGTAATTTGGAAGACTAAGCAAGAATTCAGATCTAGATACTCCTTTCCACTAGCAGCCAACAATATTAATATTCATAGCAACGAATTTGTGATGGAATTCATCGATGCTGTTG

>*S. cerasiferum*_4

CAAACCGCGGGGATGCCTCCGCTCGCCTAAACTGCAGCACGATGGACAGAATGCTTTCTGTATGGGCTGCATTTCTTACATTTAGATCATGGTGGAATATTTGTTGAGCTTTGAAGGTAGGATCTTCCCATTTTTAGTTCTACTAATTTTTGATCACGTGTATTGCATGTCGCCCCTTTGTCATTGCTATAAAATTTCTTTATACTTAAAACCTGAGTTGTAAAATAAAAAAGAATAAAACGAAGACCATTAATTTTAGTGTTTAGCTTTTTAGCTAGAAATATGCTCCCCACGAACCGTTCAACTTTACCTTAACCCCAAATTTCCCGTAAAAAAAATTAGTCTTTTGTTAAAATAAATTTTAGTCCCCTAAAAGGACCTATTTATTTTACAAATTTGAAGGTCTTTTTCCAGGCTAATTTATGTCTAGTTAACACTTTCATAAAAATTAAGAGTAGAGTAGCATATAATGTACTGCAATAGTTGACTTGATAGTTGTAATGACTCAATTAAAATAATGCCCTTATACATTTAATCATCCTCTCTAACATGGTACAGTATAAATCAATTCTCATGTAGGAGAGGATACTTGTTAAACTTTATCCAAAATTGAAGGATATGTTTACGTATTATGCCAAAAATAATCGCTGGGTAGTAAGTCAACTGAGTGTAGTACTTTTTATTGTATCTCAATTGTTCTTTTCCCAGTTTAATTCTTGTATTACAAGGATGTATTTCGACAAAGAATTGTCTGATCTGAAAAATCGCGTTGTTCGCAGATTGCAAACACGAATATTCTGGGATGTTGCTGTTGACATCTAATTTTTACCCTCCACGGTACAAATCAATCATCGAACTTTTAATTTTAACAATTTGAAATAATTAGCTTTCGAAAACGCAAAAAATATTTTCCCAAATAAATTTCTTATTTTTTATTTTTAGGAGTTATTATTCTTAATTATTTCGAGTTGATAATATTAAAAACTCTTTAATTAATTTGTATAATTCATCGTGTAGTCAAATTAGTCAATCAAATTCAATTTAATTACTTTTAACCGACTAAAATTACAATTGAATTGGTCAATTTTAAATCAACCCAATCCACTTTTAATCCCAAACCCGGCCCACTTCCCTTTTATTTTTCAGTATAATATAATATATATGTATATATGTGGCGCGTGTCCAAGTTTTTCTTTTTTTTTTTCTCCTCATTTCCTCTCACGCGTACGCACCCACTCTCTCCCTTTTCGTTCTTCTCCTTCACCTCAACAAACCAGGCGACAACCAGCAACAAACCCGACAATCCAAGATACGAATCCGTTGAGGGAATG

>*S. incanum*_6

CAAACAATACGGTACAACTCAACTATTATTCACTTAAAGCTCTCTACAGAATGCTTTCTATATTGATTGTAGCATTACATTTAGATAATAGTGGAACATTTGTCGAGCTTTGAGGGTAGGATCTTCCTATTTTTAATTATACTAATTTTTGGTAACGTGTGTTGCATGTCAGTCCTTTGTTATTGCTATAAAGTTTCTATATACTTAAAAAAGTAAGTTTCAAAATAAAAAAATGGTAAAATGAACAGCATTAATTTTATCGTTTACATTTTTAGCTGGAAATATAGTACCCAACAATAGTTTAACTTTAAACATAAATTTTTACATGTAATAATAATTTAGTCTTTTTGTTAATATTAAATTTATAGTCACATAAAATGTACTTATTTATATTACAAATTTGAAGTGTCTTTTTTCATGCTTAAATTATGTCTAGTTTAACATTTTCATATAAATTAAGAGTAGAGTAGCATATAATGTACTTGCAATAGTTGACTTGGTAGTTGTAATTGACTCAATTAAAATAATGACATAATACATATAATCGTCCTCTTTAACATGGCACAGTATAAATCAATTGTCATGTAGAAGAGGATACTTGTTAAACTTTATCCAAAATTGAAGGATACGTTTCCGTATTATGCCAAAAATAATCACAGAGTAGTAAGTCAACTGAGTGTAATACTCTTTATTGCATTTCAATTGTTCTTTTCCCAGTTTAATTCTTGTGTTACAAGGATATATTTTGAAAAAGAATTGTCAGATCTGAAAAATCGCGTTGTTTGCAGATTGCAAACACGAATATTCTGGGATGTTGCTGTTCACATCCAATTTTTACCCTCCACGGTACAAATCAATAATCAAGCTTTTAATTTTAACAATTTGAAATAATTAGCTTTCGAAAATGCAAAAAATATTTTTCCAAATAAATTTCTTATTTTTTTATTTTTAGGAGTTATTATTCTTAATTATTTCGGGTTGATAATATTAAAAACTCTTTAATTAATTTGTATAATTCATCGTGTAGTCAAATTAGTCAATCGAATTCAATTTAATTATTTTTAATTGGCTAAAATTACAATTGAATTGGTCAATTTTAAATCCCCACCTTGAACCCATTTTCAGCTTTAATAATGGACCAATTCTTGGCTCAAATAAAGTCAACCCAACCCACTTTTAATCCCAAACCCGGCCCACTTCCCTTTTATTTTTCAGCATAATATAATATATATGTATATATGTTGCGCGTGTCCTACACGCGCCCCCAATATTTCCTTTTTTTCTCCTCATTTCCTCTCACGCGTATGCACCCACTCTCCCCCTTTTCGTTCTTCTCCTTCACCTCAACAAACCAGGCGACAACCAGCAACAAACCCGACAATCCAAGAATCTCTCCAACGTTACGATAGGCAAATTCGTACATACGTGGCTTCTCCTTAGCCTCTATGATACGAATCCGTTGAGGGAATTTCCAAGTG

>*S. linnaeanum*

CAAACAATACGGTACAACTCAACTATTATTCACTTAAAGCTCTCTACAGAATGCTTTCTATATTGATTGTAGCATTACATTTAGATAATAGTGGAACATTTGTCGAGCTTTGAGGGTAGGATCTTCCTATTTTTAATTATACTAATTTTTGGTAACGTGTGTTGCATGTCAGTCCTTTGTTATTGCTATAAAGTTTCTATATACTTAAAAAAGTAAGTTTCAAAATAAAAAAATGGTAAAATGAACAGCATTAATTTTATCGTTTACATTTTTAGCTGGAAATATAGTACCCAACAATAGTTTAACTTTAAACATAAATTTTTACATGTAATAATAATTTAGTCTTTTTGTTAATATTAAATTTATAGTCACATAAAATGTACTTATTTATGTTACAAATTTGAAGTGTCTTTTTTCATGCTTAAATTATGTCTAGTTTAACACTTTCATATAAATTAAGAGTAGAGTAGCATATAATGTACTTGCAATAGTTGACTTGGTAGTTGTAATTGACTCAATTAAAATAATGACATAATACATATAATCGTCCTCTTTAACATGGCACAGTATAAATCAATTATCATGTGGAAGAGGATACTTGTTAAACTTTATCCAAAATTGAAGGATACGTTTCCGTATTATGCCAAAAATAATCACAGAGTAGTAAGTCAACTGAGTGTAATACTCTTTATTGCATTTCAATTGTTCTTTTCCCAGTTTAATTCTTGTGTTACAAGGATATATTTTGAAAAAGAATTGTCTGATCTGAAAAATCGCGTTGTTTGCAGATTGCAAACACGAATATTCTGGGATGTTGCTGTTCACATCCAATTTTTACCCTCCACGGTACAAATCAATAATCAAGCTTTTAATTTTAACAATTTGAAATAATTAGCTTTCGAAAATGCAAAAAATATTTTTCCAAATAAATTTCTTATTTTTTTATTTTTAGGAGTTATTATTCTTAATTATTTCGGGTTGATAATATTAAAAACTCTTTAATTAATTTGTATAATTCATCGTGTAGTCAAATTAGTCAATCGAATTCAATTTAATTATTTTTAATTGGCTAAAATTACAATTGAATTGGTCAATTTTAAATCCCCACCTTGAACCCATTTTCAGCTTTAATAATGGACCAATTCTTGGCTCAAATAAAGTCAACCCAACCCACTTTCAATCCCAAACCCGGCCCACTTCCCTTTTATTTTTCAGCATAATATAATATATATGTATATATGTTGCGCGTGTCCTACACGCGCCCCCAATATTTCCTTTTTTTCTCCTCATTTCCTCTCACGCGTATGCACCCACTCTCCCCCTTTTCGTTCTTCTCCTTCACCTCAACAAACCAGGCGACAACCAGCAACAAACCCGACAATCCAAGAATCTCTCCAACGTTACGATAGGCAAATTCGTACATACGTGGCTTCTCCTTAGCCTCTATGATACGAATCCGTTGAGGGAATTTCCAAGTG

>*S. lichtensteinii*

CAAACAATACGGTACAACTCAACTATTATTCACTTAAAGCTCTGTACAGAATGCTTTCTATATTGATTGTAGCATTACATTTAGATAATAGTGGAATATTTGTCGAGCTTTGAGGGTAGGATCTTCCTATTTTTAGTTATACTAATTTTTGGTAATGTGTATTGCATGTCAGTCCTTTGTTATTGCTATAAAATTTCTTTATACTTAAAAAAGTAAGTTTCAAAATAAAAAATGGTAAAATGAACAGTATTAATTTTATCGTTTACATTTTTAGCTAGAAATATAGTACCCAACAATAGTTTAACTTTAAACATCAAATTTTACATGTAATAATAATTTAGTCTTTTTGTTAAAATTAAATTTATAGTCACATAAAATGTACTTATTTATATTACAAATTTGAAGTGTCTTTTTTCATGCTTAAATTATGCTTAGTTTAACACTCTCATATAAATTAAGAGTAGAGTAGCATATAATGTACTTGCAATAGTTGACTTGATAGTTGTAATTGACTCAATTAAAATAATGACATAATGCATATAATCGTCCTCTCTAACATGGCACAGTATAAATCAATTGTCATGTAGAAGAGGATACTTGTTAAACTTTATCCAAAATTGAAGGATACGTTTACGTATTATGCCAAAAATAATCGCAGAGTAGTAAGTCAATTAAGTGTAATATTCTTTATTGTATCTCAATTGTTCTTTTTCCAGTTTAATTCTTGTGTTACAAGGATGTATTTCGACAAAGAATTGTTTGATCTGAAAAATCGCGTTGTTCGCTGCAAACACGAATATTCTGGGGTGTTGTTGTTGACATCCAATTTTTACCCTCCATGGTACAAATCAATCATCGAGCTTTTAGTTTTAACAATTTAAAATAATTAGCTTTCGAAAGTGCAAAAAATATTTTCCCAAATAAATTTCTTATTTTTTATTTTTAGGAGTTATTATTCTTAATTATTTCGGATTGATAATATTAAAAACTCTTTAGTTAATTTGTATAATTTATCGTGTAGTCAAATTAGTCAATCGAATTCAATTTGGTTACTTTTAATTGACTAAAATTGCAATTGAATTGGTCAATTTTAAATTCTCACCTTGAACTCATTTTCAGCTTTCATAATGGACCAATTTTTGACCCAAATCTAGTCAATCCAACCCACTTTTAATCTCAAACCCGGCCCATTTTCCTTTTATTTTTCAGCATAATATAATATATATGTCTATATGTGGCGCGTGTCCCCCAAKGTTTCCTTTTTTTTTTTCTACTCATTTCCTCTCATGCGTACGCACCCACTCTCTCCCTTTTCGTTCTTCTCCTTCACCTCAACAAATCAGGCGACAACCAGCAACAAACCCGACAATCCAAGAATCTCTCCAACGTTACTATAGCCAAATTCGTACATACGTGGCTTCTCCTTAGCCTCTAATTTCCAAGTG

>*S. campylacanthum*

CAAACAATACGGTACAACTCAACTATTATTCACTTAAAACTCTGTACAGAATGCTTTCTATATTGATTGTAGCATTACATTTAGATAATAGTGGAATATTTGTCGAGCTTTGAGGGTAGGATCTTCCTATTTTTAGTTATACTAATTTTTGGTAATGTGTATTGCATGTCAGTCCTTTGTTATTGCTATAAAATTTCTATATACTTAAAAAAGTAAGTTTCAAAATAAAAAATAGTAAAATGAACAGTATTAATTTTATCGTTTACATTTTTAGCTAGAAATATAGTACCCAGCAATAATTTAACTTTAAACATCAAATTTTACATGTAATAATAGTTTAGTCTTTTTGTTAAAATTAAATTTATAGTCACATAAAATGTACTTATTTATATTACAAATTGGAAGTGTCTTTTTTCGTGCTTAAATTATGTCTAGTTTAACACTTTCATATAAATTAAGAGTAGAGTAGCTATAATGTACTTGCAATAGTTGACTTGGTAGTTGTAATTGACTCAATTAAAATAATGACATAATACGTATAATCGTCCTCTCTAACATGGCACAGTATAGAATCAATTGTCATGTAGAAGAGGATACTTGTTAAACTTTATCCAAGATTGAAGGATACGTTTACGTATTATGCCAAAAATAATCGCAGAGTAGTAAGTCAATTGAGTGTAATATTCTTTATTGTATCTCAATTGTTCTTTTCCCAGTTTAATTCTTGTGTTACAAGGACGTATTTCGACAAAGAATTGTCTGGTCTGAAAAATCGCGTTGTTCGCAAATTGCAAACACGAATATTCGGGGATGTTGCTGTTGACATTCAATTTTTACCCTCCACGGTACAAATCAATCATCGAGCTTTTAGTTTTAACAATTTAAATAATTACCTTTCGAAAATGCAAAAAATATTTTTCCAAATAAATTTCTTATTTTTTATTTTTAGGAGTGATTATTCTTAATTATTTCGGATTGATAATATTAAAAACTCTTTAAGTAATTTGTATAATTTATCGTGTAGTCAAATTAGTCAATTGAATTCAATTTTACTTTTAATTGGCTAAAATTGCAATTGAATTGGTCAATTTTAAATTCTCACCTTGAACCCATTTTCAGCTTTCATAATGGACCAATTCTTGGCCCAAATCTAGTCAATCCAACCCATTTTTAATCTCAAACCCGGCCCACTTCCCTTTTATTTTTCAGCATAATATAATATATATGTATATATGTGGCGCGTGTAGGACACGTGCCCCCAATTTTTCCTTTTTTCTCCTTATTTCCTCTCACGCGTACGCACCCACTCTCTCCCTTTTCGTTCTTCTCCTTCACCTCAACAAACCAGGCGACAACCAGCAACAAACTCGATATTCCAAGAATCTCTCCAACGTTACGATAGCCAAATTCGTACATACATGGCTTCTCCTTAGCCTCTATGATACGAATCCGTTGAGGGAATTTCCAAGTG

>*S. insanum*_10

CAAACAATACGGTACAACTCAACTATTATTCACTTAAAACTCTGTACAGAATGCTTTCTATATTGATTGTAGCATTACATTTAGATGATAGTGGAATATTTGTCGAGCTTTGAGGGTAGGATCTTCCTATTTTTAGTTATACTAATTTTTGGTAATGTGTATTGCATGTCAGTCCTTTGTTATTGCTATAAAATTTCTATATACTTAAAAAAGTAAGTTTCAAAATAAAAAATAGTAAAATGAACAGTATTAATTTTATCGTTTACATTTTTAGCTAGAAATATAGTACCCAGCAATAATTTAACTTTAAACATCAAATTTTACATGTAATAATAGTTTAGTCTTTTTGTTAAAATTAAATTTATAGTCACATAAAATGTACTTATTTATATTACAAATTGGAAGTGTCTTTTTTCGTGCTTAAATTATGTCTAGTTTAACACTTTCATATAAATTAAGAGTAGAGTAGCATATAATGTACTTGCAATAGTTGACTTGGTAGTTGTAATTGACTCAATTAAAATAATGACATAATACATATAATCGTCCTCTCTAACACGACACAGTATAAAATCAATTGTCATGTAGAGGATGCTTGTTAAACTTTATCCAAAATTGAAGGATACGTTTATGTATTATGCCAAAAATAATCGCAGAGTAGTAAGTCAATTGAGTGTAATATTCTTTATTGTATCTCAATTGTTCTTTTCCCAGTTTAATTTTTGTGTTACAAGGATGTATTTCGACAAAGAATTGTCTGATCTGAAAAATTGCGTTGTTCGCAAATTGCAAACACGAATATTCGGGGGTGTTGCTGTTGACATTCAATTTTTACCCTCCACGGTACAAATCGATCATCGAGCTTTTAGTTTTAACAATTTAAATAATTACCTTTCGAAAATGCAAAAAATATTTTTCCAAATAAATTTCTTATTTTTTATTTTTAGGAGTGATTATTCTTAATTATTTCGTATTGATAATATTAAAAACTCTTTAAGTAATTTGTATAATTTATCGTGTAGTCAAATTAGTCAATTGAATTCAATTTTACTTTTAATTGGCTAAAATTGAAATTGAATTGGTCAATTTTAAATTCTCACCTTGAACCCATTTTCAGCTTTCATAACGGACAATTCTTGGCCCAAATCAGTCAATCCAACCCATTTTTAATCTCAAACCCGGCCCACTTCCCTTTTATTTTTCAGCATAATATAATATATATGTATATCTGTGGCGCGTGTAGGACACGCGCCCCCAATTTTTCCTTTTTTCTCCTTATTTCCTCTCACGCGTACGCACCTACTCTCTCCCTTTTCGTTCTTCTCCTTCACCTCAACAAACCAGGCGACAACTAGCAACAAACTCGATAATCCAAGAATCTCTCCAACGTTACGATAGCCAAATTCGTACATACATGGCTTCTCCTTAGCCTCTATGATACGAATCCGTTGAGGGAATTTCCAAGTG

>*S. melongena*

CAAACAATACGGTACAACTCAACTATTATTCACTTAAAACTCTGTACAGAATGCTTTCTATATTGATTGTAGCATTACATTTAGATAATAGTGGAATATTTGTCGAGCTTTGAGGGTAGGATCTTCCTATTTTTAGTTATACTAATTTTTGGTAATGTGTATTGCATGTCAGTCCTTTGTTATTGCTATAAAATTTCTATATACTTAAAAAAGTAAGTTTCAAAATAAAAAATAGTAAAATGAACAGTATTAATTTTATCGTTTACATTTTTAGCTAGAAATATAGTACCCAGCAATAATTTAACTTTAAACATCAAATTTTACATGTAATAATAGTTTAGTCTTTTTGTTAAAATTAAATTTATAGTCACATAAAATGTACTTATTTATATTACAAATTGGAAGTGTCTTTTTTCGTGCTTAAATTATGTCTAGTTTAACACTTTCATATAAATTAAGAGTAGAGTAGCATATAATGTACTTGCAATAGTTGACTTGGTAGTTGTAATTGACTCAATTAAAATAATGACATAATACATATAATCGTCCTCTCTAACATGGCACAGTATAAAATCAATTGTCATGTAGAAGAGGATACTTGTTAAACTTTATCCAAAATTGAAGGATACGTTTATGTATTATGCCAAAAATAATCGCAGAGTAGTAAGTCAATTGAGTGTAATATTCTTTATTGTATCTCAATTGTTCTTTTCCCAGTTTAATTCTTGTGTTACAAGGATGTATTTCGACAAAGAATTGTCTGATCTGAAAAATTGCGTTGTTCGCAAATTGCAAACACGAATATTCGGGGATGTTGCTGTTGACATTCAATTTTTACCCTCCACGGTACAAATCAATCATCGAGCTTTTAGTTTTAACAATTTAAATAATTACCTTTCGAAAATGCAAAAAATATTTTTCCAAATAAATTTCTTATTTTTTATTTTTAGGAGTGATTATTCTTAATTATTTCGTATTGATAATATTAAAAACTCTTTAAGTAATTTGTATAATTTATCGTGTAGTCAAATTAGTCAATTGAATTCAATTTTACTTTTAATTGGCTAAAATTGAAATTGAATTGGTCAATTTTAAATTCTCACCTTGAACCCATTTTCAGCTTTCATAATGGACCAATTCTTGGCCCAAATCTAGTCAATCCAACCCATTTTTAATCTCAAACCCGGCCCACTTCCCTTTTATTTTTCAGCATAATATAATATATATGTATATCTGTGGCGCGTGTAGGACACGCGCCCCCAATTTTTCCTTTTTTCTCCTTATTTCCTCTCACGCGTACGCACCCACTCTCTCCCTTTTCGTTCTTCTCCTTCACCTCAACAAACCAGGCGACAACTAGCAACAAACTCGATAATCCAAGAATCTCTCCAACGTTACGATAGCCAAATTCGTACATACATGGCTTCTCCTTAGCCTCTATGATACGAATCCGTTGAGGGAATTTCCAAGTG

# Legends to Supplementary Figures

**Supplementary Figure 1**: Expression profiles of co-transcripts in accessions of *S. melongena* (green)*, S. incanum* (red) and *S. insanum* (grey). FPKM: Fragments per kilobase million. Dotted line shows the expression level of ORC6, used as a constitutively expressed control.

**Supplementary Figure 2**: BEAST tree showing placement of BBVA, BBVB and GBV in genus Badnavirus. Bayesian inference analysis was performed on the RT-RNase H domains corresponding to positions 5599-6882 in the genome of Commelina yellow mosaic virus (NC_001343), the type member of genus Badnavirus. All nodes have PP ≥ 0.90 and bootstrap values are indicated on the nodes. Sequences of the proposed new species are shown in light red box.

**Supplementary Figure 3:** PCR amplification of the V*R1* locus and its left border from *Solanum* spp.

(A) Representation of the VR1 locus of *S. melongena* showing placement of the primers used for PCR experiments and expected amplification products. Amplification products obtained from *Solanum* spp. with (B) or without (C) BBVA insertion using primer pair InlF2 / InlR2. (D): Amplification products obtained from *Solanum* spp. with BBVA insertions using primer pair InfL2/R1U.

1: *S. incanum* (accession number in INRAE's germplasm collection: MM00684); 2 : *S. incanum* (MM00711); 3: *S. insanum* (MM00669); 4: *S. insanum* (MM00675); 5: *S. insanum* (MM00686); 6: *S. lichtensteinii* (MM01248); 7: *S. linnaeanum* (MM00195); 8: *S. melongena* (MM00609); 9: *S. melongena* (MM01290); 10: *S. melongena,* (MM01547), 11: *S. melongena* (MM01712), 12: *S. melongena* (MM01791), 13: *S. melongena* ( MM10439); 14: *S. melongena* (MM12137); 15: *S. ovigerum* (MM0290); 16: *S. umtuma*  (MM12212); 17: *S. campylacanthum* (MM00679); 18: *S. rigidum* (MM01696); 19: *S. cerasiferum* (MM01389). C: Amplification products obtained from *Solanum* spp. without BBVA insertion using primer pair InlF2 / InlR2. 20: *S. aethiopicum* (MM00943); 21: *S . anguivi* (MM12366); 22: *S. dasyphyllum* (MM10240), 23: *S. macrocarpon* (MM00150); 24: *S. richardii* (MM00869); 25: *S. trilobatum* (MM01025), 26: *S. violaceum* (MM01027).
