# Supplementary figures and images for: Insertion of Badnaviral DNA in the Late Blight Resistance Gene (R1a) of Brinjal Eggplant (Solanum melongena)
Source: Front Plant Sci. 2021 Jul 23;12:683681. doi: 10.3389/fpls.2021.683681 (PMC8346255; doi:10.3389/fpls.2021.683681)

## Slide 1
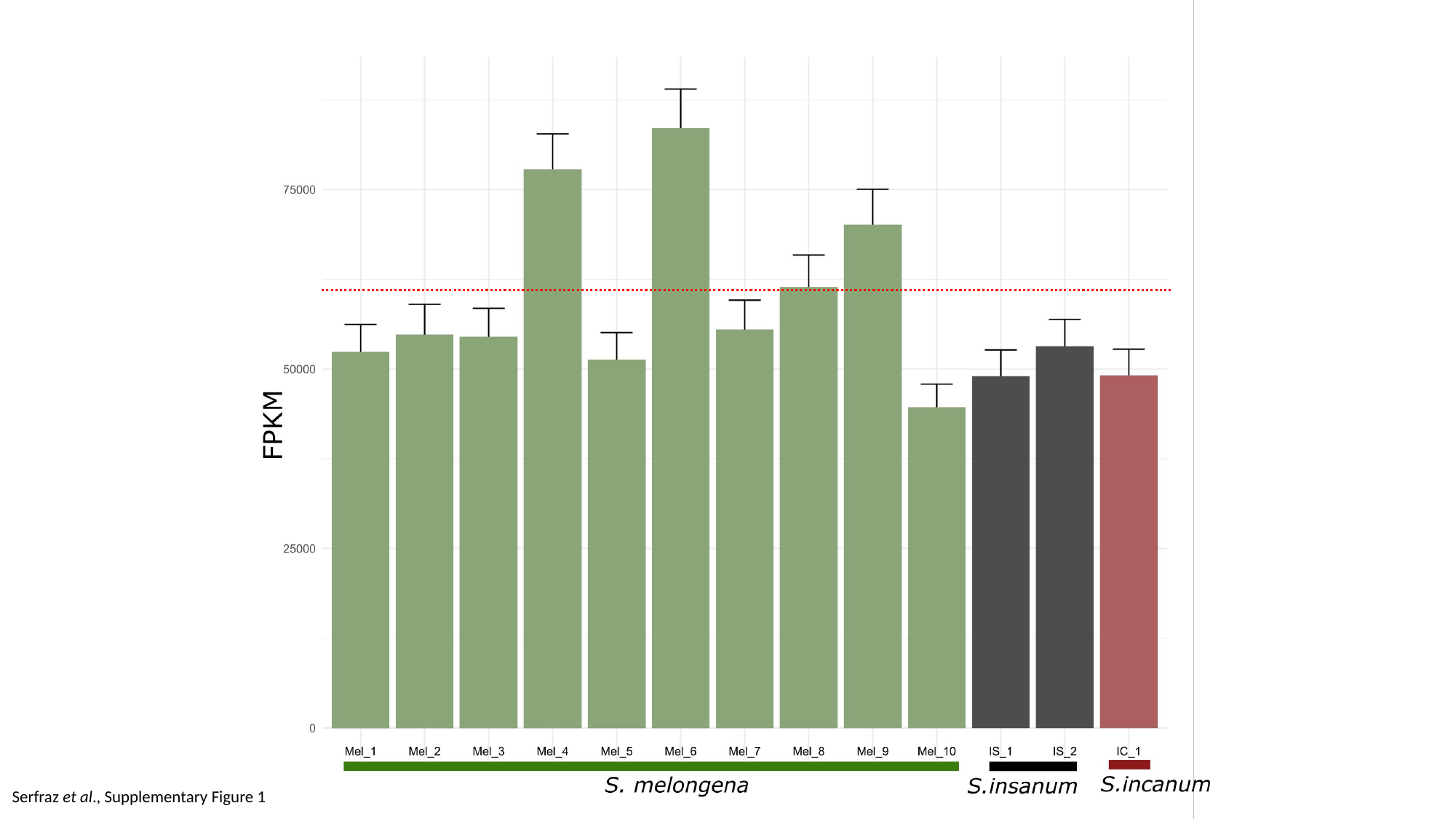

Serfraz et al., Supplementary Figure 1

Supplement: Supplementary file 1 [file Data_Sheet_1.zip › Supplementary Figure 1.pptx]

## Slide 1
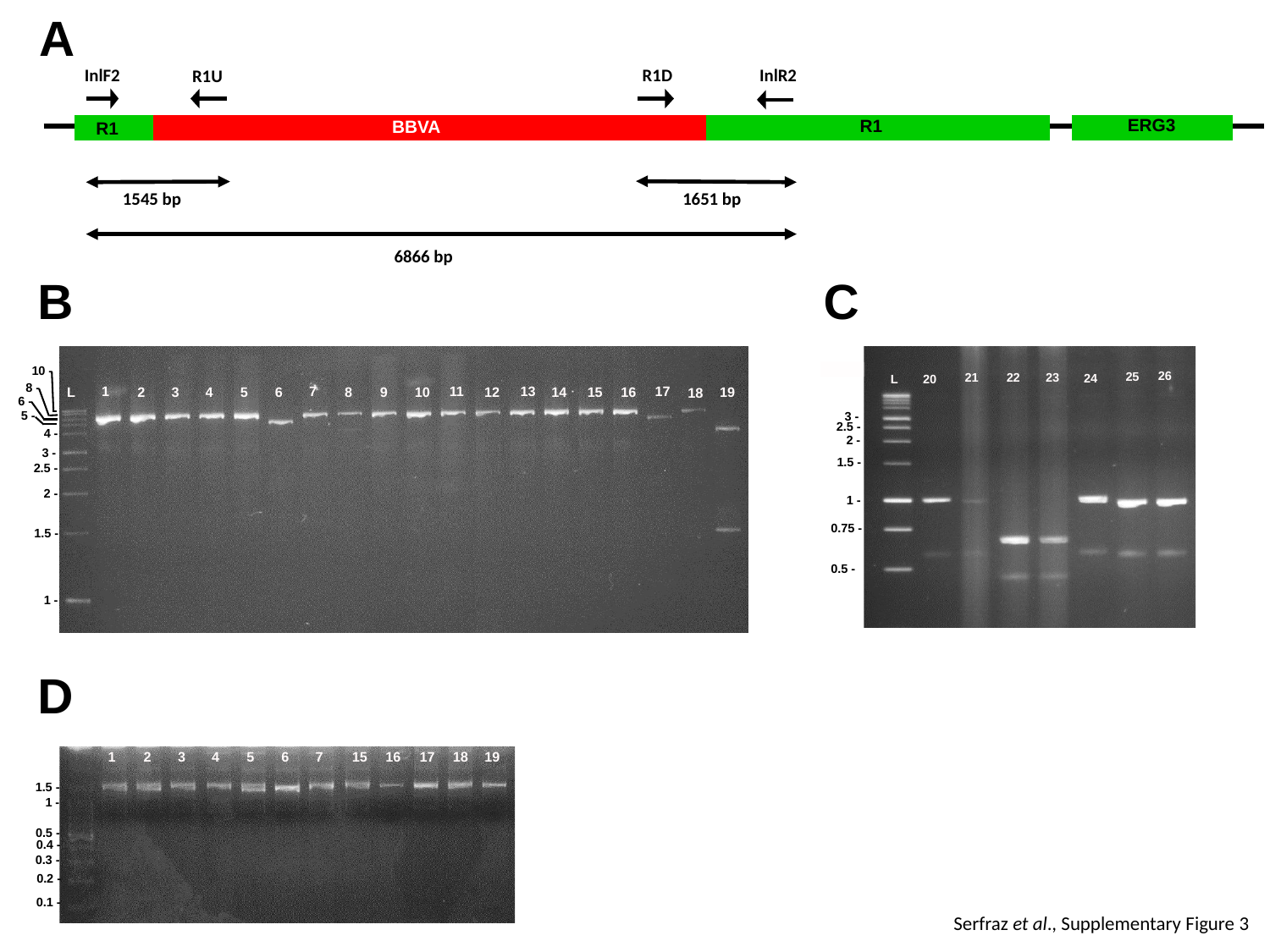

A
InlF2
R1D
InlR2
R1U
ERG3
R1
BBVA
R1
1545 bp
1651 bp
6866 bp
B
10 -
8 -
17
1
7
11
13
2
3
4
16
5
12
15
8
19
9
14
L
6
10
18
6 -
5 -
4 -
3 -
2.5 -
2 -
1.5 -
1 -
C
26
25
23
21
22
24
20
L
3 -
2.5 -
2 -
1.5 -
1 -
0.75 -
0.5 -
D
18
1
2
3
4
5
6
7
15
16
17
19
1.5 -
1 -
0.5 -
0.4 -
0.3 -
0.2 -
0.1 -
Serfraz et al., Supplementary Figure 3

Supplement: Supplementary file 1 [file Data_Sheet_1.zip › Supplementary Figure 3.pptx]
